# Integrated transcriptome and small RNA sequencing in revealing miRNA-mediated regulatory network of floral bud break in *Prunus mume*

**DOI:** 10.3389/fpls.2022.931454

**Published:** 2022-07-22

**Authors:** Man Zhang, Wenhui Cheng, Xi Yuan, Jia Wang, Tangren Cheng, Qixiang Zhang

**Affiliations:** Beijing Key Laboratory of Ornamental Plants Germplasm Innovation and Molecular Breeding, Beijing Laboratory of Urban and Rural Ecological Environment, Key Laboratory of Genetics and Breeding in Forest Trees and Ornamental Plants of Ministry of Education, National Engineering Research Center for Floriculture, School of Landscape Architecture, Beijing Forestry University, Beijing, China

**Keywords:** transcriptome analysis, small RNA sequencing, co-expression network, floral bud break, dormancy release, *Prunus mume*

## Abstract

MicroRNAs is one class of small non-coding RNAs that play important roles in plant growth and development. Though miRNAs and their target genes have been widely studied in many plant species, their functional roles in floral bud break and dormancy release in woody perennials is still unclear. In this study, we applied transcriptome and small RNA sequencing together to systematically explore the transcriptional and post-transcriptional regulation of floral bud break in *P. mume*. Through expression profiling, we identified a few candidate genes and miRNAs during different developmental stage transitions. In total, we characterized 1,553 DEGs associated with endodormancy release and 2,084 DEGs associated with bud flush. Additionally, we identified 48 known miRNAs and 53 novel miRNAs targeting genes enriched in biological processes such as floral organ morphogenesis and hormone signaling transudation. We further validated the regulatory relationship between differentially expressed miRNAs and their target genes combining computational prediction, degradome sequencing, and expression pattern analysis. Finally, we integrated weighted gene co-expression analysis and constructed miRNA-mRNA regulatory networks mediating floral bud flushing competency. In general, our study revealed the miRNA-mediated networks in modulating floral bud break in *P. mume*. The findings will contribute to the comprehensive understanding of miRNA-mediated regulatory mechanism governing floral bud break and dormancy cycling in wood perennials.

## Introduction

Bud dormancy is an important adaptive strategy for perennial plant species to survive harsh environmental conditions (Rohde and Bhalerao, 2007). Perennial trees in temperate regions went through seasonal cycling of growth and dormancy to preserve vegetative or reproductive primordium during harsh winters (Yang et al., 2021). Bud dormancy can be classified into three categories: paradormancy, endodormancy, and ecodormancy (Lang et al., 1987). Paradormancy is known as growth inhibition controlled by internal hormones and organ competition. Endodormancy is defined as inhibited bud growth that can only be overcome by chilling temperatures. Bud endodormancy is usually triggered by shortening photoperiod and/or low temperature (Cooke et al., 2012; van der Schoot et al., 2013). After accumulating sufficient chilling, endodormant buds enter ecodormancy state and acquire competence to resume vegetative growth or flowering in suitable conditions (Rinne et al., 2011; van der Schoot et al., 2013). Unlike annual or biennial plants, floral initiation and blooming is interposed by bud dormancy (Ramos et al., 2018; Hsiang et al., 2021). In *Prunus* species, floral induction started in late summer with reproductive organs differentiating and developing before dormancy period (Julian et al., 2011). When the chilling requirement is fulfilled, the arrested floral tissues continue to grow and mature, followed by the subsequent production of gametes and blooming in the following spring (Lloret et al., 2018). In the background of global climate change, warm winters and irregular weather changes have been disrupting important phenological events, such as timing of bud break and flowering (Hatfield and Prueger, 2015). Insufficient chilling in floral buds can lead to erratical flowering, deformed flowers, and reduced fruit set (Luedeling, 2012; Ladwig et al., 2019). Therefore, it is important to understand the regulation mechanism underlying floral bud break and flowering in perennial trees.

Many recent studies have been conducted in elucidating the molecular control of floral bud dormancy cycling in deciduous fruit trees, such as pear, peach, apple (Yang et al., 2021). Till now, a large number of transcription factors were characterized in regulating floral bud dormancy and development. For example, DAM (DORMANCY ASSOCIATED MADS-BOX) transcription factors were firstly identified in the *evergrowing* peach mutants and were found essential for dormancy establishment and maintenance (Yamane et al., 2011). The over-expression of *DAM* genes led to delayed bud break in apple (Rongmei et al., 2017). CBFs (C-repeat binding factors) is a group of transcription factors that regulate *DAM* genes by binding to their promoter regions upon cold induction (Niu et al., 2016). In peach, the TCP (TEOSINTE BRANCHED1/CYCLOIDEA/PROLIFERATING CELL FACTOR) transcription factor, TCP20 can interact with ABF2 (ABSCISIC ACID RESPONSIVE ELEMENTS-BINDING FACTOR 2) and promote dormancy release by repressing *DAM5* and *DAM6* expression (Leubner et al., 2020). Additionally, HD-ZIP family transcription factors are also involved in regulating bud dormancy. For example, HB22 (HOMEOBOX PROTEIN 22) regulates *DAM6* expression by binding the promoter of *DAM1* in pear (Yang et al., 2018). Epigenetic modifications also participate in regulating bud dormancy transition. It is reported that the modified level of H3K27 trimethylation and H3K4 trimethylation were associated with *DAM* gene transcription during bud break (Zhu et al., 2020). Despite the advancements in understanding transcriptional regulation, how post-transcriptional mechanism is involved in regulating floral bud break in temperate perennials still remains elusive.

MicroRNAs (miRNAs) is a class of endogenous, small non-coding RNA molecules of 18–24 nucleotides that can modulate gene expression post-transcriptionally in both plants and animals (Rubio-Somoza and Weigel, 2011; Spanudakis and Jackson, 2014). MiRNAs bind to the coding region or UTR region of target genes through near-perfect base complementation and induced target gene silencing by endonucleolytic cleavage in plants or translational inhibition in animals (Yu et al., 2017). MiRNAs was first transcribed by RNA polymerase II into primary miRNAs (pri-miRNA), which were cropped into the stem-loop structured precursors (pre-miRNAs), exported to cytoplasm by exportin, and subsequently cleaved by cytoplasmic RNase III Dicer, generating miRNA duplex (Kim, 2004; Lee et al., 2004; Strzyz, 2021). The unwinding duplex was further incorporated into AGO proteins to assemble RISC (RNA-induced silencing complex) that targets mRNA for cleavage or translation repression (Betancur et al., 2012; Strzyz, 2021). In plants, miRNAs play a central role in many developmental processes including patterning of leaf and flower organs, timing of phase transition, seed development, biotic and abiotic responses through targeting transcriptional factor encoding genes (D’Ario et al., 2017; Fang and Wang, 2021). The role of miRNAs in chilling-induced dormancy release has been investigated in a few woody perennials (Yu et al., 2021). In *Pyrus pyrifolia*, miRNA6390 was found to target *DAM* gene transcripts for degradation and promote dormancy release (Niu et al., 2016). MiR160, involved in auxin signaling pathway, was found significantly induced after chilling treatment in dormant cambium tissues in poplar (Ding et al., 2014). In tea tree, miR169, miR408, miR414, and miR782 were found up-regulated and miR828, miR1864, miR852, and miR1425 were down-regulated as vegetative buds exit dormancy (Jeyaraj et al., 2014). A recent study of ncRNAs (non-coding RNAs) during dormancy transition in peach identified two essential miRNAs, miR6286 targeting ASPARAGINE-RICH PROTEIN encoding gene involved in abscisic acid signaling and miR2275 that regulates anther microsporogenesis at the stage of dormancy release (Yu et al., 2021). So far, the molecular function of miRNAs during floral bud dormancy transition has not been fully examined yet.

*Prunus mume* Sieb. et Zucc., also known as Japanese apricot, is one of the world’s important perennial fruit crops (Wu et al., 2019). *P. mume* was first originated from southern China and has a domestication history over 1,000 years (Gao et al., 2012). The fruits of *P. mume* have many health-beneficial physiochemicals and can be processed into beverage, jams, or used for culinary purposes (Kim et al., 2018; Bailly, 2020). Moreover, being one of the earliest flowering woody perennial, *P. mume* encompass a great number of varieties and has been widely used for ornamental and landscape purposes (Zhang Q. et al., 2018). Despite extensive efforts in understanding the genetic control of bud break and blooming in *P. mume*, the molecular network is far from complete (Zhao et al., 2018a,b; Zhang et al., 2021b). The knowledge of dormancy release regulation in *P. mume* will facilitate our understanding into the post-transcriptional regulation of bud dormancy cycling in temperate tree species (Zhang et al., 2021b). In this study, we applied transcriptome and small RNA sequencing analysis to analyze the global expression change of mRNAs and miRNAs during floral bud break in *P. mume*. By combining computational prediction and degradome sequencing, we validated the regulatory relationship between miRNAs and their targeting genes. With weighted co-expression analysis, we screened out a number of miRNA-mRNA regulatory pairs and constructed the miRNA mediated regulatory networks that associated with chilling-induced floral bud break in *P. mume*. In general, our study provided novel candidate microRNAs and regulatory genes for future functional investigations.

## Materials and methods

### Dormancy status evaluation

Floral bud phenology was surveyed for *P. mume* cultivar “Subaitaige” grown in Jiufeng International Germplasm Garden (Beijing, China) from October until full bloom during 2020–2021. The air temperature was recorded hourly since October in 2021. To assess the dormancy status of floral bud, we performed bud break competency tests every 2 weeks following the method described by Ionescu et al. (2017). One-year old shoots with at least 10 lateral floral buds were collected every 2 weeks, immersed in water, and incubated in the phytotron under forcing conditions (16 h day/8 h night, 22°C/20°C, 70% relative humidity). The basal part of all branches was cut every week to avoid xylem clogging and the water was changed every 2 days. Bud break rate (BBR) was measured as the percentage of floral buds that flushed after 2 weeks incubation. To evaluate the chilling requirement, we determined the time point of endodormancy release as the date when 50% of all floral buds become competent to flush under forcing conditions (Hsiang et al., 2021). Chilling hours was calculated based on the chilling hour model (0∼7.2°C) to measure the progression of chilling accumulation.

### Sample collection

Floral buds were collected at four developmental stages (endodormancy I, endodormancy II, ecodormancy, and bud flush) with three biological replicates during bud break process. Samples were instantly frozen in liquid nitrogen and total RNA was extracted from the flower buds using Trizol reagent kit (Invitrogen, Carlsbad, CA, United States) according to the manufacturer’s protocol. The quantity and purity of total RNA was assessed with Agilent 2100 Bioanalyzer (Agilent Technologies, Palo Alto, CA, United States) and verified on 1% RNase free agarose gel.

### Transcriptome sequencing and analysis

The sample mRNA was enriched with Oligo (dT) beads, reverse transcribed, and prepared to construct cDNA libraries. Twelve sequencing libraries were sequenced on the Illumina HiSeq™ 2500 platform in paired-end mode (Gene Denovo Biotechnology Co., Guangzhou, China). Raw reads were obtained and low quality reads were filtered with software fastp (version 0.18.0) (Chen et al., 2018). The raw data was uploaded into the NCBI Sequence Read Archive (accession number: PRJNA833165). Clean paired-end reads were aligned to the genome of *P. mume* (Zheng et al., 2021) with HISAT2 (Kim et al., 2015). Based on the alignment, transcript abundance was estimated using StringTie v1.3.1 (Pertea et al., 2015, 2016). Read counts were normalized to FPKM value (fragment per kilobase of transcript per million mapped reads) using RSEM software (Li and Dewey, 2011). Differentially expressed genes (DEGs) were defined as genes with | log2FC| > 1.5 and adjusted *P* < 0.05 in each two sample stage comparison using DESeq R package (Love et al., 2014). The expression patterns of the DEGs were visualized with “pheatmap” R package^1^.

### Small RNA sequencing and analysis

Small RNAs of 18–30 nucleotides were isolated from total RNA, reverse-transcribed, and amplified by PCR. Twelve small-RNA sequencing libraries were constructed following recommended protocol and were sequenced on Illumina HiSeq™ 2500 platform by Gene denovo Biotechnology Co., Ltd. (Guangzhou, China). Finally, the sequencing data was deposited in the NCBI Sequence Read Archive (accession number: PRJNA832606).

Raw reads were filtered to remove low quality reads (reads containing bases with Q < 20, reads with more than 5% N, reads with contaminated 5′ adaptor, reads without 3′ adaptor, or reads with polyA tail) and adaptors were trimmed off using software Fastx-toolkit (Gordon and Hannon, 2010). Clean reads were compared with small RNAs in GeneBank database v236.0 (Benson et al., 2007) and Rfam database v14.0 (Griffiths-Jones, 2004) to remove sequence tags annotated as ncRNAs (rRNA, scRNA, snoRNA, snRNA, and tRNA). Additionally, we aligned the clean reads to the genome of *P. mume* to remove fragments that mapped to exons, introns, or repeated sequences using SOAP v2.20 (Li R. et al., 2009). The remaining tags were searched against miRBase v22.1 database to identify known miRNAs (Griffiths-Jones, 2006). The miRNAs with less than two mismatches or those with at least 16 bp overlap with known miRNAs were assigned to the same miRNA family (Goldstien et al., 2010). Unannotated tags were used to identify novel miRNAs based on their hairpin structures with software miRDeep2 (Friedländer et al., 2012) following standard protocols (Min and Yoon, 2010). The newly identified miRNAs and their precursor sequences were folded into secondary structure and were visualized with RNAfold^2^.

The transcription level of miRNA was estimated and normalized to TPM (transcripts per million) value following the formula:


(1) $$TPM=\frac{\mathrm{actual}\mathrm{miRNA}\mathrm{counts}}{\mathrm{total}\mathrm{counts}\mathrm{of}\mathrm{clean}\mathrm{reads}}\times 10{}^{6}$$

The expression levels of known and novel miRNAs were compared between adjacent developmental stages using DESeq2 software. Differentially expressed miRNAs (DEmiRs) were identified as miRNAs with log_2_(FC) ≥ 1 and FDR corrected *p*-value < 0.05. To examine the expression patterns of DEmiRs, we visualized the TPM values of DEmiRs using “pheatmap” R package.

### MicroRNA-target gene identification

The target genes of miRNAs were predicted by searching against *P. mume* reference genome using software Patmatch (Yan et al., 2005) based on the following criteria: (1) MFE (minimum free energy) of miRNA-target duplex ≥ 74%; (2) mismatches between the sRNA and their targets (G-U as 0.5 mismatch) ≤ 4; (3) adjacent mismatches allowed in miRNA-target duplex ≤ 2; (4) no adjacent mismatches allowed in position 2–12 of miRNA-target duplex; (5) no mismatches allowed in position 10–11 of miRNA-target duplex; (6) mismatches allowed in position 2–12 of miRNA-target duplex ≤ 2.5. To validate the miRNA-mRNA relationship, equal amounts of RNA from all samples were pooled to construct degradome sequencing library. The cleaved RNA products were ligated with adaptors, reverse transcribed and sequenced on Illumina HiSeq™ 2500 platform. Raw reads from degradome library were processed in a similar fashion as described above and the clean reads annotated as ncRNAs were removed. The remaining clean tags were analyzed with CleaveLand pipeline v3.0 to predict miRNA cleavage sites. Potential target genes were identified as those with alignment score ≤ 4 and no mismatches between 10^th^ and 11^th^ nucleotides of miRNAs. The target genes were classified into five categories based on raw tag sequence abundance within cleavage sites and those within category 0–2 were kept as true targets (Yu et al., 2021). The raw degradome sequencing data was available from NCBI Sequence Read Archive (accession number: PRJNA832060).

### Co-expression network analysis of mRNA and miRNAs

To identify co-regulatory genes or miRNAs that associated with the progression of bud dormancy release, we performed weighted gene co-expression network analysis (WGCNA) on all genes and miRNAs separately with WGCNA (v1.47) package in R. WGCNA is a scale-free network construction approach that finds clusters of genes with highly correlated expression profiles (Langfelder and Horvath, 2008). For the gene network construction, we first removed lowly expressed genes and kept only genes with FPKM ≥ 2 across 70% samples and SD > 0.25. Then we estimated the Pearson’s correlation coefficients among genes based on the FPKM values. The correlation matrix was converted into adjacency matrix (soft threshold power = 9). Subsequently, we transformed the adjacency matrix into topological overlap measures (TOM) and performed hierarchical clustering to group all genes into clusters using Dynamic Tree Cut algorithm. Similarly, the weighted co-expression network of miRNAs was constructed to group all miRNAs (TPM ≥ 2 across 70% samples; SD > 0.25) into associated modules following standard WGCNA protocols (soft-threshold power = 8). All genes or miRNAs were assigned to co-expressed modules that were named with colors. The minimum module size was set as 50 and the threshold of the module similarity was set at 0.8. Using bud break rate as phenotype, we identified most relevant modules of genes or miRNAs as candidate regulators determining dormancy status during floral bud break. The hub genes or miRNAs were selected as those with top 0.1% module membership for each relevant module.

### MiRNA-mRNA regulatory network construction

To investigate the miRNA-mRNA regulatory mechanism during floral bud break, we performed an integrative analysis on the miRNA and mRNA co-expression networks. We mapped all DEGs and the target genes of DEmiRs identified in previous sections to the co-expressed gene network with their correlations integrated. Differentially expressed transcription factors (TFs) and hub genes were identified from the trait-related gene modules and the miRNA-TF gene pairs were added to the miRNA-mRNA network. Finally, the network was visualized by Cytoscape software (version 3.7.2) (Shannon et al., 2003).

### Functional enrichment analysis

To detect the biological function of candidate genes, we performed functional enrichment analysis on the DEG sets and target gene sets of DEmiRs. Genes were mapped to Gene Ontology (GO) terms of biological progress (BP), molecular function (MF), and cell component (CC) in the GO database (Gene Ontology Consortium, 2004). GO terms with FDR corrected *p*-values < 0.05 were considered as significantly enriched in hypergeometric tests (Yan et al., 2020). *P*-values were calculated as follows:


P=1-∑i=0m-1(Mi)(N-Mn-i)(Nn)

Where N is the total number of background genes, n is the number of candidate genes in N, M is the total number of genes annotated to the GO term, and m is the number of candidate genes in M. The *p*-values were adjusted with FDR (false discovery rate) and the GO terms with adjusted *p*-values ≤ 0.05 were considered as significantly enriched among the candidate genes. To understand the metabolic or signal transduction pathways, we also mapped candidate gene sets to the KEGG (Kyoto Encyclopedia of Genes and Genomes) database and considered pathways with FDR ≤ 0.05 as significantly enriched following the same hypergeometric tests.

### Expression pattern analysis with quantitative real-time PCR

To validate the expression pattern of miRNAs and their target genes, we performed quantitative real-time PCR (qRT-PCR) assays. We first extracted RNA from the same samples with Plant RNA Kit (Omega Bio-Tek, United States) and reverse transcribed using PrimeScript RT Kit (Takara Bio, Japan). The qRT-PCR assays of candidate genes were conducted using TB Green^®^ Premix Ex Taq™ II (Takara Bio, Japan) on CFX96 Real-Time PCR Detection System (Bio-Rad Laboratories, Inc., United States) following protocol: 95°C for 5 min, followed by 40 cycles at 95°C for 5 s, 60°C for 30 s and 72°C for 30 s. The relative gene expression level was computed with 2^–ΔΔ*Ct*^ method using *PP2A* as the reference gene for candidate genes. Small RNA was extracted with Plant miRNA Kit (Omega, China) following the manufacturer’s protocols. Mature miRNAs were reverse transcribed into cDNA using Mir-X™ miRNA First-Strand Synthesis Kit (Code No. 638315, Takara, China). The qPCR was carried out using miRNA SYBR Green RT-qPCR Kit (Takara, China) following similar temperature settings. The relative expression level of miRNA was estimated using 5S rRNA as internal reference. Primer pairs of candidate genes and miRNAs were designed with NCBI primer-blast and were provided (Supplementary Table 1).

## Results

### Physiological changes during floral bud break

**FIGURE 1:**
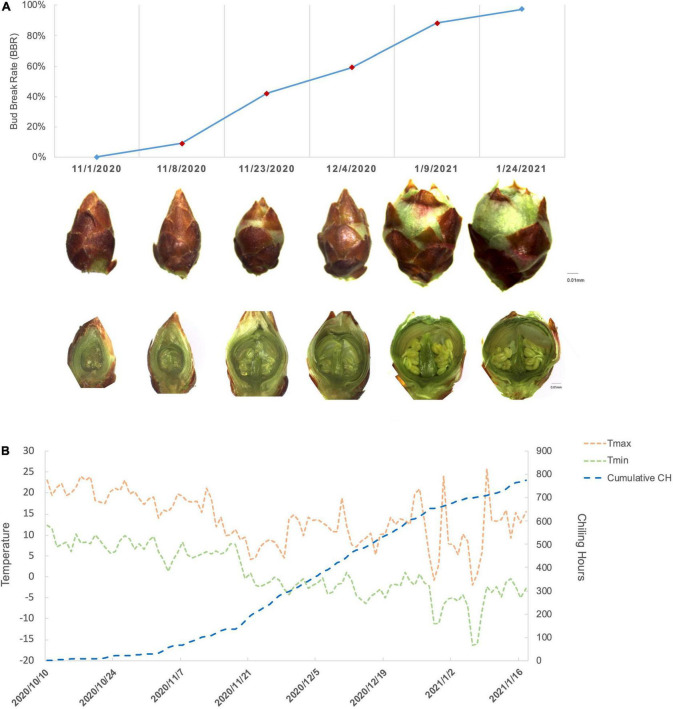
The characterization of floral bud development for *P. mume* cultivar “Subaitaige” during dormancy release in 2020–2021. **(A)** The floral bud dormancy status and inner structure changes of floral bud sampled during dormancy release and bud flush. The longitudinal sections of flower buds were compared between sampling time points. **(B)** The daily maximum temperature, minimum temperature, and cumulative chilling hours were evaluated since October 10th, 2020 till January 24th, 2021 using chilling hour model.

During the progression of floral bud break, we tracked the morphological changes of *P. mume* “Subaitaige” and evaluated the bud break rate as an indicator of dormancy depth from dormancy induction to bud flush during 2020–2021 (Figure 1A). Based on bud dormancy competency test, we estimated the date of endodormancy break to be between November 29th and October 12th, 2020 and we observed 5% blooming on February 10th, 2021 (Figure 1A). Using the chilling hour model, we calculated total chilling hours (CH) required for cultivar “Subaitage” as approximately 320 CHs, which started to accumulate since October 12th, 2020 (Figure 1B). In *P. mume*, floral organ initiation was induced in late summer with floral primordium continuing to develop throughout winter (Zhang et al., 2021a). In early November, floral buds were maintained in endodormant state with floral organ primordiums, including sepal, petal, stamen, and pistil fully differentiated. During the chilling accumulation process, endodormant flower buds continued to develop. From late December to January, green sepals were revealed, floral bud enlarged rapidly with floral organs, such as stamen and ovule matured before natural bud flush, suggesting the complete flower organ differentiation after dormancy release (Figure 1A). To understand the molecular dynamics during dormancy transition, we selected samples of four time points corresponding to floral buds of average bud break percentage at 9.1%, 42.1%, 59.02%, and 88.3% for the following analysis (Figure 1B). We termed the four sampling stages as endodormancy I (Endodor I), endodormancy II (Endodor II), ecodormancy (Ecodor), and bud flush (Bflush) on the basis of bud morphology and chilling accumulation.

### Transcriptome analysis and differentially expressed gene identification

To identify genes that participate in regulating bud dormancy release, we performed RNA-seq analysis on floral bud tissues of four developmental stages. We constructed 12 sequencing libraries and obtained a total of 80.3 Gb raw reads. After quality control, we generated 79.5 Gb clean reads with total mapping rate between 93 and 94% across samples (Table 1). Principal component analysis (PCA) revealed that the first two principal components explained 47.8 and 33.2% of total variation (Supplementary Figure 1A). Pearson correlation among samples indicated that samples of similar physiological state are more closely related (Supplementary Figure 1B). These results confirmed that the transcriptional state is consistent with physiological differences among flower bud samples.

**TABLE 1:** Summary table of the transcriptome sequencing data of four developmental stages.

Sample	Raw read (bp)	Clean read (bp)	Q20 (%)	Q30 (%)	GC (%)	Unique mapped (%)	Total mapped (%)
Endodor I_1	609,2395,800	6,047,021,936	98.26	94.71	44.87	90.4	94.20
Endodor I_2	7,625,247,300	7,557,165,051	98.16	94.46	44.89	90.41	94.24
Endodor I_3	5,455,417,500	5,398,187,733	98.00	94.18	44.94	89.89	93.66
Endodor II_1	5,651,884,200	5,593,496,800	97.88	93.97	44.82	90.5	94.24
Endodor II_2	6,135,953,400	6,086,968,752	98.36	94.96	44.73	91.18	94.93
Endodor II_3	7,696,955,100	7,609,774,831	97.86	93.83	45.18	90.35	94.19
Ecodor_1	6,883,355,100	6,782,062,169	97.88	93.96	45.15	90.45	94.19
Ecodor_2	6,590,915,400	6,532,084,067	98.33	94.85	45.09	91.15	94.99
Ecodor_3	5,974,257,900	5,908,850,440	98.04	94.24	45.04	90.74	94.50
BFlush_1	6,523,971,300	6,442,412,281	97.95	94.08	44.86	90.4	94.25
BFlush_2	8,199,327,900	8,143,543,358	97.77	93.59	45.17	90.78	94.74
BFlush_3	7,458,137,700	736,6106,977	97.93	94.07	45.18	90.2	94.11

**FIGURE 2:**
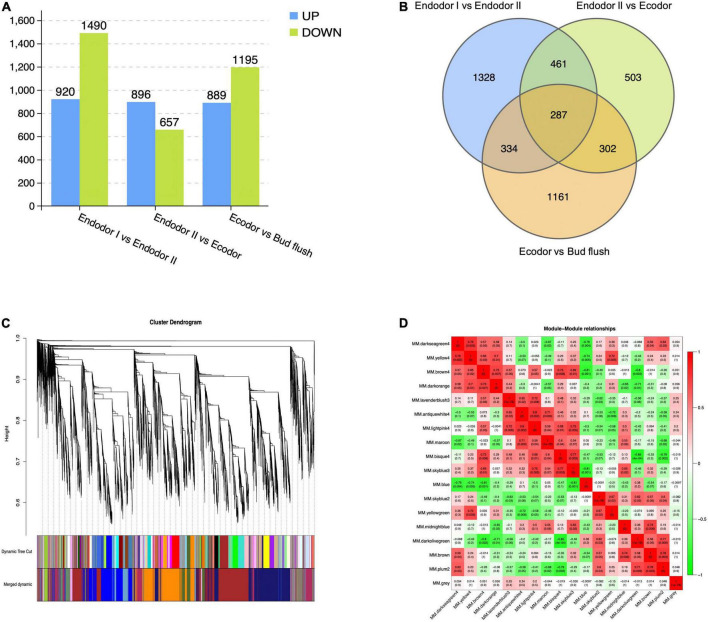
The identification of differentially expressed genes and co-expressed gene modules from WGCNA analysis. **(A)** The number of up-regulated or down-regulated DEGs among stage comparisons (Endodor I vs. Endodor II, Endodor II vs. Ecodor, and Ecodor-vs.-Bud flush). **(B)** Venn diagram of DEGs identified across stage comparisons (Endodor I vs. Endodor II, Endodor II vs. Ecodor, and Ecodor-vs.-Bud flush). **(C)** Hierarchical clustering dendrogram showing 17 co-expressed gene modules in designated colors from WGCNA analysis. **(D)** Heatmaps showing Pearson correlation among eigengenes of co-expressed gene modules. The Pearson correlation coefficients was colored according to the score.

Differential expression analyses were performed by comparing gene expression between adjacent developmental stages. Among 30,082 genes investigated, we detected 2,410 genes differentially expressed between endodormancy stage I and II (Figure 2A). These include 920 up-regulated and 1,490 down-regulated DEGs (Figure 2A). Between endodormancy and ecodormancy, a total of 1,553 DEGs were detected including 896 up-regulated and 657 down-regulated genes (Figure 2A). Additionally, 2,084 DEGs were identified in the comparison between ecodormancy and bud flush stage (Figure 2A). By comparing the DEGs between stage comparisons, we identified 1,328, 503, and 1,161 DEGs that are specific to the comparison endodor I vs. endodor II, endodor II vs. ecodor, and ecodor-vs.-bud flush, respectively (Figure 2B). A total of 287 DEGs were shared by all three stage comparisons (Figure 2B).

### Functional enrichment analysis of differentially expressed genes

To further investigate the function of DEGs, we performed GO and KEGG pathway enrichment analysis on the DEG sets. The DEGs between endodormancy stage I and II are significantly enriched in biological processes including response to carbohydrate stimulus, response to ethylene stimulus, response to abscisic acid stimulus, response to cold, and response to gibberellin stimulus (Supplementary Table 2). KEGG enrichment analysis confirmed that biosynthesis of secondary metabolites, carbohydrate metabolism, and DNA replication are three most enriched pathways (Supplementary Figure 2A). The DEGs during the transition from endodormancy to ecodormancy were mainly participated in reproductive developmental process, response to temperature stimulus, and anatomical structure development (Supplementary Table 2). The KEGG pathways including photosynthesis, DNA replication, and carbon fixation were significantly enriched (Supplementary Figure 2B). Finally, DEGs between ecodormancy and bud flush were mostly involved in biological processes including DNA replication, flower development, gametophyte development, and response to hormone stimulus (Supplementary Table 2). Pathways such as DNA replication, phenylpropanoid biosynthesis, flavonoid biosynthesis, and plant hormone signal transduction are mostly over-represented in this stage comparison (Supplementary Figure 2C). These results illustrated the important biological processes and metabolic pathways of these DEGs during dormancy transition process in *P. mume*.

### Co-expression network analysis of mRNA

**FIGURE 3:**
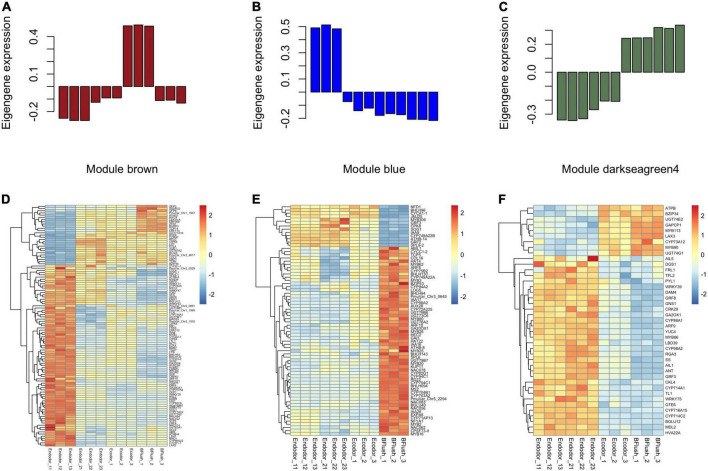
The co-expressed gene modules associated with dormancy transition in the WGCNA analysis. **(A–C)** The barplot displaying the normalized eigengene expression for module brown **(A)**, module blue **(B)**, and module darkseagreen4 **(C)**. **(D–F)** The heatmaps showing the expression pattern of differentially expressed genes within module brown **(D)**, module blue **(E)**, and module darkseagreen4 **(F)**.

To identify the co-regulatory factors during floral bud break in *P. mume*, we performed weighted gene co-expression network analysis (WGCNA) and grouped 15,381 genes into 17 co-expressed modules with their pairwise correlation evaluated (Figures 2C,D). Among all modules, brown module contained the maximum number of genes (3,259 genes), while the yellow module contained 64 genes (Figures 2C,D). To further characterize the gene modules essential to the progression of floral bud development, we estimated the correlation between module eigengenes and bud break rate. We identified three gene modules, including module blue, module brown, and module darkseagreen4 that were significantly associated with bud flush rate (Supplementary Table 3 and Figure 3). The eigengene of module blue was positively correlated with BBR (*r* = 0.81, *p*-value = 0.002) (Supplementary Table 3 and Figure 3). On the other hand, eigengenes of module brown (*r* = −0.91, *p*-value = 4e^–5^) and darkseagreen4 (*r* = −0.84, *p*-value = 7e^–4^) were negatively correlated with BBR (Supplementary Table 3 and Figure 3).

The eigengene of module brown increased gradually as dormancy depth decreased during chilling accumulation but significantly dropped during bud flush (Figure 3A). Genes within module brown were mostly involved in biological processes such as reproductive organ development, meristem development, and response to dsRNA. The brown module genes annotated to floral organ development included *EMS1* (EXCESS MICROSPOROCYTES1) that encodes a basic leucine zipper (bZIP) transcription factor regulating microsporogenesis (Zhao et al., 2002), E-class MADS-box transcription factor SEP3 (SEPALLATA3) (Immink et al., 2009), and LUG (LEUNIG), a transcriptional repressor regulating gynoecium and ovule development in *Arabidopsis* (Liu et al., 2000; Supplementary Table 4). Among the DEGs, we detected a few transcription factors that are known to be associated with bud dormancy control (Supplementary Table 4). For example, the transcript levels of *DAM6* and *SVP* significantly decreased as chilling accumulates (Figure 3D). SVP (SHORT VEGETATIVE PHASE), a MADS-box transcription factor, acts as a floral repressor in *Arabidopsis* (Wang et al., 2018). In poplar, SVP-like genes can integrate the short-photoperiod and low-temperature induced endodormancy pathways to promote ABA signaling and bud dormancy establishment (Singh et al., 2018). *SOC1* displayed decreasing expression pattern in the early stage of endodormancy release (Figure 3D). SOC1 is another MADS-box transcription factor acting downstream of *FT* (FLOWERING LOCUS T) in promoting flowering in *Arabidopsis* (Lee and Lee, 2010). In *Prunus avium*, SOC1 can interact with DAM5 *in vivo*, suggesting their interaction is required for floral bud dormancy regulation in trees (Wang et al., 2020). We also identified a few chilling inducible genes, such as the *CBF4* (C-REPEAT/DRE BINDING FACTOR 4), dehydrin protein COR47 (COLD-REGULATED 47), and *RCI2A* (RARE-COLD-INDUCIBLE 2A). CBF4 is a member of ERF/AP2 transcription factor induced by cold temperatures and can activate the downstream dehydrin COR family proteins, such as COR47, through binding to the C-repeat core regulatory motif (Welling and Palva, 2008). The over-expression of *CBF4* in *Arabidopsis* leads to higher tolerance to cold and drought (Haake et al., 2002). In perennial trees, CBFs, with their expression triggered by cold, can induce the transcription of *DAM5* and *DAM6* to establish dormancy (Niu et al., 2016; Zhao et al., 2018a).

Module blue is consisted of 2,064 genes with its eigengene significantly decreased during the dormancy transition period (Figure 3B). The GO enrichment analysis showed that genes within module blue are mostly involved in biological processes including anatomical structure development, gametophyte development, and pollen development. DEGs related to female gametophyte development within blue module include *NFD1* (NUCLEAR FUSION DEFECTIVE 1) and CPSF73-II (CLEAVAGE AND POLYADENYLATION SPECIFICITY FACTOR 73 KDA SUBUNIT-II) (Supplementary Table 5). The pollen development associated DEGs include *CYP703A2* (CYTOCHROME P450, FAMILY 703, SUBFAMILY A, POLYPEPTIDE 2), *MS2* (MALE STERILITY 2), and *KUP11* (K+ UPTAKE PERMEASE 11) (Supplementary Table 5). These gametophyte development related genes were significantly up-regulated after endodormancy release and maintained high expression level during bud flush (Figure 3E). A few differentially expressed transcription factors from module blue were detected. For example, *ATHB-8* (ARABIDOPSIS THALIANA HOMEOBOX 8) and *ATHB-14* encode HD-ZIP (homeodomain leucine zipper) transcription factors that are known to regulate SAM (shoot apical meristem) and vascular patterning in *Arabidopsis* (Ramachandran et al., 2017). Two MYB transcription factors, *MYB5* (MYB DOMAIN PROTEIN 5) that possibly involved in the formation of endosperm layers (Li S. F. et al., 2009) and *MYB26* regulating anther dehiscence were found differentially expressed (Yang et al., 2017; Supplementary Table 5).

Genes within module darkseagreen4 are mostly annotated to biological processes including anatomical structure development, response to stress, and regulation of cell size. The eigengene of module darkseagreen4 dramatically increased during the progression of bud dormancy break (Figure 3C). Among the darkseagreen4 module genes, we identified a few AP2 family transcription factors that may putatively regulate floral bud break, such as *ANT* (AINTEGUMENTA) required for cell proliferation control during bud break in poplar, *AIL1* (AINTEGUMENTA-LIKE 1) and *AIL5* with partially overlapping functions with *ANT* in flower development functions (Krizek, 2015; Supplementary Table 6). These genes were highly expressed during endodormancy release but were decreased slightly afterward, suggesting their role in floral organ development (Figure 3F). Two GRFs, *GRF5* (GROWTH-REGULATING FACTOR 5) and *GRF8*, both encode transcriptional activators of pistil development were also identified in module darkseagreen4 (Liang et al., 2013; Supplementary Table 6). These two genes were highly expressed in dormant floral buds but then dropped after complete floral organ development (Figure 3F).

### Small RNA sequencing of floral bud

**FIGURE 4:**
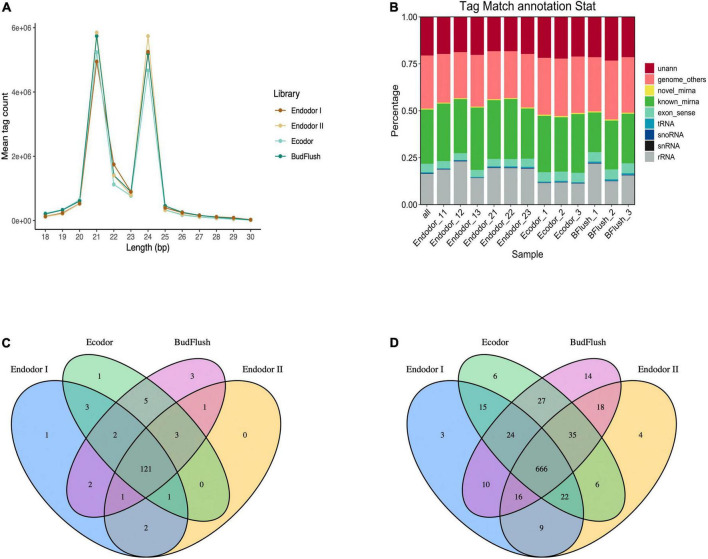
Overview of small RNA sequencing analysis. **(A)** The size distribution of small RNAs generated across four developmental stages. **(B)** The annotation of clean sequence tags across 12 small RNA sequencing libraries of four developmental stages. **(C,D)** The Venn diagram of known miRNAs **(C)** and novel miRNAs **(D)** identified across four developmental stages.

To study the functional role of miRNAs in bud break process, we constructed 12 small RNA sequencing libraries using the same samples representing four different dormancy stages and obtained approximately 15.6 million raw reads for each library (Table 2). An average of 14.9 million clean tags (95.24%) were retained after removing low quality reads and the unique tags were used for downstream analysis (Table 2). Among all libraries, the length of small RNAs ranged from 18 to 30 nt with two abundance peaks at 21 and 24 nt (Figure 4A). Small RNAs of 21 and 24 nt were mostly enriched accounting for 36.7 and 35.1% on average among total clean tags, respectively (Figure 4A). The small RNAs were first annotated by blasting against NCBI GenBank and Rfam databases to annotate non-coding RNAs. Among all annotated tags, 19.34–23.75, 20.12–20.43%, 11.97–12.67%, and 16.73–22.92% were annotated as non-coding RNAs for endodormancy I, endodormancy II, ecodormancy, and bud-flush samples, respectively (Figure 4B and Supplementary Table 7). All clean tags were also mapped to the reference genome of *P. mume* (Zheng et al., 2021) with mapping rate ranging from 72.1 to 75.9% across 12 sequencing libraries (Supplementary Table 7).

**TABLE 2:** Summary statistics of sequence reads generated from 12 small RNA sequencing libraries.

Sample	Total raw reads	High quality reads (%)	Missing 3′ adapter reads (%)	Missing insert reads (%)	5′ adapter contaminant reads (%)	Clean tags (%)
Endodor I_1	14,873,210	99.39	0.07	0.34	0.21	91.81
Endodor I_2	13,214,501	99.36	0.08	0.33	0.22	92.10
Endodor I_3	16,065,852	99.49	0.08	0.29	0.13	96.55
Endodor II_1	14,120,322	99.35	0.10	0.31	0.20	94.16
Endodor II_2	16,628,062	99.39	0.20	0.37	0.22	94.16
Endodor II_3	18,261,367	99.44	0.12	0.36	0.17	95.88
Ecodor_1	18,514,077	98.84	0.13	0.29	0.09	96.14
Ecodor_2	15,378,330	99.38	0.19	0.29	0.08	96.95
Ecodor_3	14,192,577	99.41	0.06	0.28	0.09	96.47
BFlush_1	15,738,502	99.36	0.05	0.39	0.18	95.94
BFlush_2	15,201,731	99.32	0.24	0.32	0.10	96.06
BFlush_3	15,207,626	99.42	0.06	0.36	0.11	96.63

### Known and novel mRNA identification

After excluding ncRNAs and reads mapped to exons, introns or repeat sequences, we annotated the remaining small RNAs by blasting against miRBase 22.0 database (Kozomara et al., 2019). In total, we identified 146 known miRNAs, among which 121 miRNAs were present in floral bud tissues of all developmental stages (Figure 4C and Supplementary Table 8). Mostly of the known miRNAs were present among 12 floral bud samples with only few miRNAs specific to certain developmental stages. For example, miR11544-z was specific to endodormancy I stage, miR7782-y specific to ecodormancy stage, and miR6294-z, miR5083-z, miR172-x specific for samples of bud-flush stage, respectively (Figure 4C). The known miRNAs were mostly 21 nt in length with hairpin structures ranging from 80 to 366 nt (Supplementary Table 8). Additionally, 875 novel miRNAs were identified using Mireap software with secondary structures predicted with mirDeep2 (Figure 4D). Of all novel miRNAs, 666 miRNAs were shared across all sequencing libraries (Figure 4D).

### Differential expression analysis of microRNAs

**FIGURE 5:**
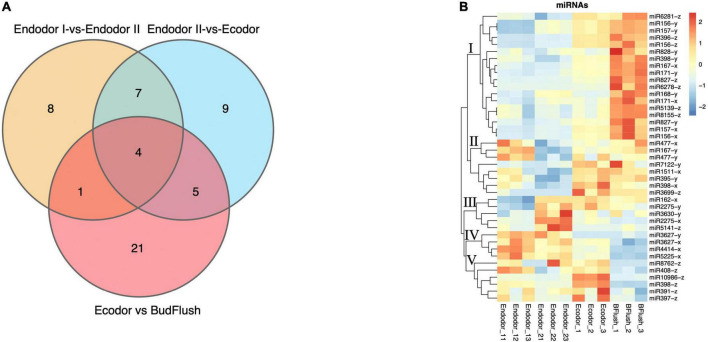
The differential expression analysis identified known miRNAs differentially expressed across stage comparisons. **(A)** Venn diagram of known DEmiRNAs identified across four stage comparisons. **(B)** Expression heatmap of known miRNAs displaying their expression pattern across four developmental stages.

In order to identify differentially expressed miRNAs (DEmiRs) during floral bud break in *P. mume*, we compared the normalized expression of miRNAs between every two adjacent stages. By summing up all DEmiRs, we identified 41 known miRNAs and 53 novel miRNAs that were significantly differentially expressed (Figures 5A,B and Supplementary Figures 3A,B). Among them, 63, 83, and 93 DEmiRs were detected in the comparison of endodormancy I-vs.-endodormancy II, endodormancy II-vs.-ecodormancy, and ecodormancy-vs.-bud flush, respectively (Figure 5A and Supplementary Figure 3A). Among the DEmiRs, four known miRNAs (miR156-x, miR157-x, miR2275-x, and miR408-z) and eight novel miRNAs were common to all three comparisons (Figure 5A and Supplementary Figure 3A). We performed hierarchical clustering analysis and grouped the 41 known DEmiRs into five major clusters based on their expression profiles (Figure 5B). Cluster I contained 18 known DEmiRs, including miR157-x/y, miR156-x/y, miR396-z, and miR828-y that were highly expressed in flushed floral buds (Figure 5B). In cluster II, DEmiRs such as miR167-y, miR398-x, miR477-x were significantly induced in floral buds after endo-dormancy release (Figure 5B). Cluster III is consisted of a few miRNAs (such as miR162-x, miR2275-x/y, miR3630-y) that were highly expressed in stage endodormancy II but their expression decreased afterward (Figure 5B). The fourth group included miR3627-x/y, miR4414-x, and miR5225-x, whose expression remained high in endo-dormant floral buds but decreased as floral bud exit dormancy. Known DEmiRs within cluster V showed mixed expression pattern during the process of dormancy break (Figure 5B).

### Target gene identification of differentially expressed miRNAs

To understand the functions of differentially expressed miRNAs, we performed degradome sequencing on sample pools and obtained 22,942,638 clean reads for subsequent analysis (Supplementary Table 9). By intersecting the target genes predicted computationally and those supported with degradome data, we identified 80 valid target genes for 33 known miRNAs, most of which are transcription factors (Table 3). For example, miR164 family, which targets NAC domain transcription factors, was found to target *NAC021* (PmuVar_Chr3_1567) and *NAC098* (PmuVar_Chr3_1908) for degradation (Table 3). Similarly, miR166 target three HD-ZIP transcription factors including *REV*, *ATHB-8*, and *ATHB-14* (Table 3).

**TABLE 3:** Target genes of known miRNAs validated with degradome sequencing.

miRNA	Target	Cleave site	Score	Degradome category	Gene name	Description
miR156-z	PmuVar_Chr1_1472	802	2	2	SPL9-1	Squamosa promoter-binding-like protein 9
miR156-z	PmuVar_Chr4_1953	943	1	2	SPL13	Squamosa promoter-binding-like protein 13
miR156-z	PmuVar_Chr6_1159	260	4	2	MEAF6	Chromatin modification-related protein MEAF6
miR156-z	PmuVar_Chr8_1993	805	2	2	SPL9-2	Squamosa promoter-binding-like protein 9
miR157-x	PmuVar_Chr1_1472	802	1	2	SPL9-1	Squamosa promoter-binding-like protein 9
miR157-x	PmuVar_Chr8_1993	805	1	2	SPL9-2	Squamosa promoter-binding-like protein 9
miR159-y	PmuVar_Chr1_2929	571	3	1	−	
miR159-y	PmuVar_Chr3_1846	742	2	0	−	Rho GTPase-activating protein gacII-like
miR159-y	PmuVar_Chr5_0699	970	2.5	0	GAM1	GAMYB-like transcription factor
miR159-y	PmuVar_Chr8_1741	133	4.5	1	SAUR45	SAUR-like auxin-responsive protein family
miR160-x	PmuVar_Chr1_1276	1,370	0.5	0	ARF18-1	Auxin response factor 18-like
miR160-x	PmuVar_Chr1_3619	1,355	1	0	ARF18-2	Auxin response factor 18-like
miR160-x	PmuVar_Chr2_0764	1,373	0.5	0	ARF17	Auxin response factor 17
miR160-x	PmuVar_Chr5_2526	1,340	1	0	ARF16	Auxin response factor 18-like
miR161-y	PmuVar_Chr3_2081	571	2	1	−	
miR162-y	PmuVar_Chr5_2395	3,231	1	0	DCL1	Dicer-like 1
miR164-x	PmuVar_Chr3_1567	709	2	0	NAC021	NAC domain-containing protein 21/22
miR164-x	PmuVar_Chr3_1908	730	2	0	NAC098	Protein CUP-SHAPED COTYLEDON 2
miR166-y	PmuVar_Chr1_3612	565	2.5	0	REV	Homeobox-leucine zipper protein REVOLUTA
miR166-y	PmuVar_Chr2_1302	574	2.5	0	ATHB-8	Homeobox-leucine zipper protein ATHB-8
miR166-y	PmuVar_Chr3_0935	589	2.5	0	ATHB-14	Homeobox-leucine zipper protein ATHB-14
miR168-x	PmuVar_Chr7_1597	478	0	0	AGO1A	Protein argonaute 1-like [Prunus mume]
miR171-y	PmuVar_Chr1_1062	989	4.5	0	BCAT5	Chloroplast branched-chain amino acid aminotransferase
miR171-y	PmuVar_Chr2_0548	437	4.5	1	PUR5	Phosphoribosyl-aminoimidazole synthetase
miR171-y	PmuVar_Chr5_1720	902	2.5	0	SCL6-1	GRAS family transcription factor
miR171-y	PmuVar_Chr7_2476	1,415	2.5	0	SCL6-2	GRAS family transcription factor
miR172-x	PmuVar_Chr1_2565	1,514	3	3	−	RNA binding family protein
miR172-y	PmuVar_Chr1_1236	1,477	0.5	0	AP2	APETALA 2
miR172-y	PmuVar_Chr1_1333	1,285	0.5	0	RAP2.7-1	AP2 domain containing protein
miR172-y	PmuVar_Chr5_2600	1,354	1	0	RAP2.7-2	AP2 domain containing protein
miR1863-z	PmuVar_Chr3_0756	556	4	4	−	Pentatricopeptide repeat (PPR) superfamily protein
miR1873-z	PmuVar_Chr2_2500	2,257	4	0	−	BRO1-like domain-containing protein
miR2118-z	PmuVar_Chr3_1880	148	2.5	0	−	Hypothetical protein PRUPE_4G174200 [Prunus persica]
miR319-y	PmuVar_Chr1_0202	164	2	2	YAB2	Plant-specific YABBY family protein
miR319-y	PmuVar_Chr2_0175	989	4.5	2	NUF2	Nuclear filament-containing protein 2
miR319-y	PmuVar_Chr2_0491	9,958	4.5	2	UPL1	Ubiquitin-protein ligase
miR319-y	PmuVar_Chr3_1845	1,055	2.5	0	TCP4	TCP family transcription factor 4
miR3630-y	PmuVar_Chr2_3641	1,130	3	4	ANKRD13B	Ankyrin repeat family protein
miR3630-y	PmuVar_Chr8_1181	1,088	4.5	4	CPK28	Calcium Dependent Protein Kinase
miR390-x	PmuVar_Chr3_0848	1,053	3.5	3	GH31-1	Glycosyl hydrolases family 31
miR390-x	PmuVar_Chr3_1067	1,062	3.5	3	GH31-2	Glycosyl hydrolases family 31
miR391-x	PmuVar_Chr2_2835	179	4	3	−	Ankyrin repeat family protein
miR393-x	PmuVar_Chr2_5329	147	4	1	BHLH62	Basic helix-loop-helix DNA-binding superfamily protein
miR393-x	PmuVar_Chr4_1917	1,513	1	0	−	F-box/RNI-like Superfamily protein
miR393-x	PmuVar_Chr6_0886	1,663	1.5	0	−	F-box/RNI-like superfamily protein
miR394-x	PmuVar_Chr2_3427	1,336	1	0	FBX6	F-box only protein 6
miR395-y	PmuVar_Chr2_3827	345	3.5	0	PAPSS2	Bifunctional 3-phosphoadenosine 5-phosphosulfate synthetase
miR398-z	PmuVar_Chr1_1573	71	3	1	−	
miR408-y	PmuVar_Chr8_1636	815	3	3	U2AF35A	U2 auxiliary factor small subunit
miR472-z	PmuVar_Chr5_1864	1,157	4	0	−	Protein kinase superfamily protein
miR482-y	PmuVar_Chr5_1426	623	4	0	−	NB-ARC domain-containing disease resistance protein
miR482-y	PmuVar_Chr8_2153	623	3	0	−	LRR and NB-ARC domains-containing disease resistance protein
miR482-y	PmuVar_Chr8_2159	545	3	0	−	NB-ARC domain-containing disease resistance protein
miR482-y	PmuVar_Chr8_2160	545	3	0	−	LRR and NB-ARC domains-containing disease resistance protein
miR482-y	PmuVar_Chr8_2161	623	3	0	−	LRR and NB-ARC domains-containing disease resistance protein
miR482-y	PmuVar_Chr8_2162	611	3	1	−	NB-ARC domain-containing disease resistance protein
miR482-y	PmuVar_Chr8_2164	623	3	0	−	NB-ARC domain-containing disease resistance protein
miR482-y	PmuVar_Chr8_2167	611	3	1	−	NB-ARC domain-containing disease resistance protein
miR482-y	PmuVar_Chr8_2172	611	3	1	−	LRR and NB-ARC domains-containing disease resistance protein
miR482-z	PmuVar_Chr5_0304	1,051	3	1	−	NB-ARC domain-containing disease resistance protein
miR482-z	PmuVar_Chr5_0313	769	3	0	−	NB-ARC domain-containing disease resistance protein
miR482-z	PmuVar_Chr6_0190	613	3	0	−	NB-ARC domain-containing disease resistance protein
miR5059-z	PmuVar_Chr2_4062	2,994	4.5	1	CRD1	Cellulose-related DUF810
miR5225-x	PmuVar_Chr6_2511	3,017	4	0	−	Nucleic acid binding protein
miR535-z	PmuVar_Chr1_1472	807	2.5	0	SPL9-1	Squamosa promoter binding protein-like 9
miR535-z	PmuVar_Chr2_3424	1,161	2	4	SPL2	Squamosa promoter binding protein-like 2
miR535-z	PmuVar_Chr8_1993	805	2.5	0	SPL9-2	Squamosa promoter binding protein-like 9
miR6284-z	PmuVar_Chr1_2409	318	2.5	2	−	Peptidyl-prolyl cis-trans isomerase
miR6284-z	PmuVar_Chr2_0924	1,024	4.5	2	−	S-adenosyl-L-methionine-dependent methyltransferases superfamily protein
miR6284-z	PmuVar_Chr5_0094	1,851	4.5	2	−	Subtilisin-like serine endopeptidase family protein
miR6284-z	PmuVar_Chr7_0277	2,649	4	2	RBOHA	Respiratory burst oxidase protein F
miR7122-x	PmuVar_Chr1_3431	308	4	4	−	ATP binding protein
miR7122-x	PmuVar_Chr1_3457	347	2	2	−	ATP binding protein
miR7122-x	PmuVar_Chr1_3524	347	2.5	2	−	ATP binding protein
miR7122-x	PmuVar_Chr1_3533	101	2.5	2	−	ATP binding protein
miR7122-x	PmuVar_Chr6_2596	698	2	2	−	ATP binding protein
miR828-z	PmuVar_Chr2_3132	319	2.5	0	MYB66-1	Myb domain protein 66
miR828-z	PmuVar_Chr6_0733	382	3	0	MYB12	Myb domain protein 12
miR828-z	PmuVar_Chr7_0960	319	1	0	MYB66-2	Myb domain protein 66
miR828-z	PmuVar_Chr7_2380	340	2	0	MYB2	Myb domain protein 2
miR828-z	PmuVar_Chr8_0398	379	2	0	MYB82	Myb domain protein 82
miR858-x	PmuVar_Chr2_1740	309	2.5	0	MYB3-1	Myb domain protein 3
miR858-x	PmuVar_Chr2_1742	165	2.5	0	MYB3-2	Myb domain protein 3
miR858-x	PmuVar_Chr4_1449	357	2.5	0	TT2	TT2-like myb transcription factor

With functional enrichment analysis, we detected a number of biological pathways enriched among the target genes of DEmiRs. Between two endo-dormancy stages, regulation of organ morphogenesis, single-organism transport, and fatty acid metabolic process were mostly over-represented GO terms (Supplementary Table 10). Target genes of DEmiRs between endodormancy and ecodormancy were mostly involved in lignin metabolic process, phenylpropanoid metabolic process, and regulation of organ formation (Supplementary Table 10). Finally, the DEmiRs detected between ecodormancy and bud flush stages are targeting genes related to carbohydrate transport and secondary metabolic process (Supplementary Table 10). With KEGG pathway enrichment analysis, we observed genes of metabolic pathways such as biosynthesis secondary metabolites, plant hormone signal transduction, and starch and sucrose metabolism were enriched for DEmiRs across all three stage comparisons (Supplementary Table 11).

### Relationship analysis of the expression pattern of differentially expressed miRNAs and their target genes

**FIGURE 6:**
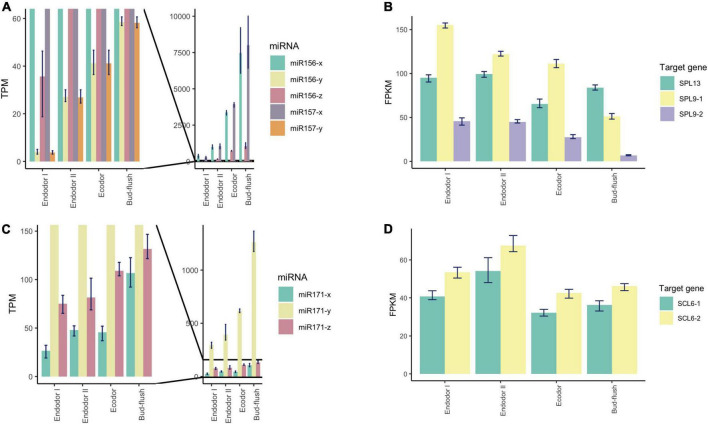
Expression pattern analysis of miR156, miR157, miR171, and their target genes during floral bud break in *P. mume*. **(A,B)** The barplots revealing the expression levels of miR156/157-x/y/z **(A)** and their target genes **(B)** during floral bud break process. **(C,D)** The barplots revealing the expression levels of miR171-x/y/z **(C)** and their target genes **(D)** during floral bud break.

To explore the regulatory mechanism of miRNAs, we analyzed the expression pattern of DEmiRs and their target genes. The Pearson correlation coefficients between the transcript level of DEmiRs and their target genes across four developmental stages ranged from −0.997 to 0.994. Among them, we observed antagonized expression patterns among many known miRNAs-TF pairs. For example, miR156 and miR157 are two miRNA families that were previously reported to regulate reproductive meristem transition and flowering time by repressing the SPL (SQUAMOSA PROMOTER BINDING PROTEIN-LIKE) transcription factors in *Arabidopsis* (Sharma et al., 2016; Millar et al., 2018). During dormancy release, the expression of miR156 and miR157 family miRNAs increased as floral bud exit dormancy, whereas their target genes including *SPL13*, *SPL9-1*, and *SPL9-2* showed decreasing expression patterns (Figures 6A,B). Among different members of the miR156-157 family, miR156-y and miR157-y were relatively low-expressed throughout the developmental process (Figure 6A). With degradome sequencing, the cleavage sites of miR156-157 targeted genes were detected within *SPL9* and *SPL13* (Supplementary Figures 4A,B). The miRNAs of miR171 family were found constantly increasing during the transition from endodormancy to bud flush. Their target genes, *SCL6-1* (SCARECROW-LIKE PROTEIN 6) and *SCL6-2*, two GARS family transcription factors were induced during endodormancy release, but their expression levels decreased slightly during bud break (Figures 6C,D).

**FIGURE 7:**
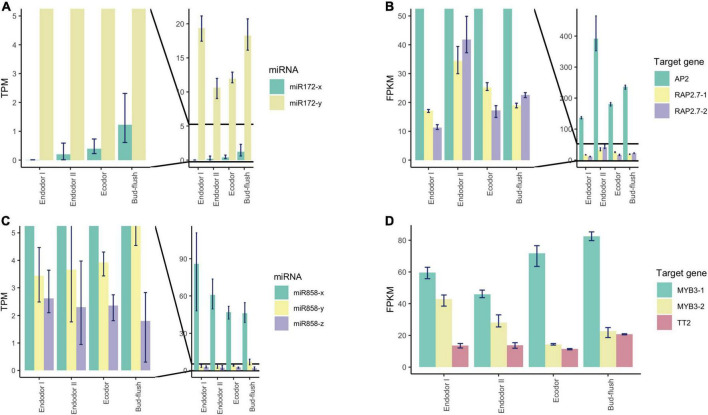
Expression pattern analysis of miR172, miR858, and their target genes during floral bud break in *P. mume*. **(A,B)** The barplots revealing the expression levels of miR172-x/y **(A)** and their target genes **(B)** during floral bud break process. **(C,D)** The barplots revealing the expression levels of miR858-x/y/z **(C)** and their target genes **(D)** during floral bud break.

Among the conserved miRNA families, miR172 is another key miRNA group that regulate age-dependent flowering timing by targeting AP2/ERF transcription factors (Wu et al., 2009). In *P. mume*, the level of miR172-y first decreased during the endodormancy transition to ecodormancy, however, was significantly increased during bud flush (Figure 7A). On the other hand, miR172-x was slightly up-regulated during bud break and was relatively low-expressed comparing to miR172-y (Figure 7A). Their target genes displayed the opposite expression patterns, where *AP2* and *RAP2.7* first increased during endodormancy release but then remained at lower level during bud flushing (Figure 7B). MiR858 was previously reported to regulate plant growth and flowering timing. The loss function of miR858 can cause early flowering, while its over-expression induces retarded plant growth and delayed flowering time (Sharma et al., 2016). In our study, miR858-x was significantly down-regulated during the floral bud break, while miR858-y slightly increased despite low expression level (Figure 7C). Among their target genes, *MYB3-1* was slightly increased, whereas the transcription level of its paralog *MYB3-2* slightly dropped during bud flushing (Figure 7D). The contrasting expression patterns suggest that different members from the same miRNA family may have divergent roles by targeting different target genes. The cleavage events of these miRNAs were verified within their target genes in the degradome analysis (Supplementary Figure 4).

The miR319 family is known to regulate TCP transcription factors, which is a class of plant-specific transcription factors that regulate cell proliferation and differentiation (Li et al., 2021). In peach, TCP20 regulate *DAM5* and *DAM6* by binding to the cis-acting element of their promoters (Wang et al., 2019). TCP18, acting downstream of *SVL* (SHORT VEGETATIVE PHASE-LIKE), is a negative regulator of vegetative bud break in poplar (Singh et al., 2018). In our study, we detected significantly decreasing transcript level of miR319-y, while miR319-x and miR319-z remained lowly expressed (Supplementary Figure 5A). Their target genes, *TCP2* and *TCP4*, on the other hand, were up-regulated first and then were down-regulated after endodormancy release (Supplementary Figure 5B). Significantly increasing transcription of miR164-x was observed during floral bud break (Supplementary Figure 5C). MiR164 was characterized to regulate root formation and drought resistance in maize and rice (Li et al., 2012; Fang et al., 2014). Their target genes *NAC021*, *NAC098*, and *NAC100* were slightly decreased during dormancy release (Supplementary Figure 5D). We also detected a few DEmiRs with no sliced target genes found in degradome analysis. For example, we observed that the expression of miR2275-x and miR2275-y were up-regulated before dormancy release, but were significantly dropped after dormancy release (Supplementary Figure 5E). While their predicted target genes (PmuVar_Chr2_1878 and PmuVar_Chr3_0920) decreased slightly and then increased significantly during bud break (Supplementary Figure 5F). It is reported that miR2275 was present in eudicot plants and are especially enriched in meiotic stages (Xia et al., 2019). In flowering plants, miR2275 can trigger the production of 24-nt phasiRNAs that regulate pollen development (Xia et al., 2019). It is likely that transcriptional change of miR2275 is associated with anther microsporogenesis in *P. mume* during floral bud break.

### Co-expression network analysis of miRNA

Weighted co-expression network analysis approach was also applied on miRNAs to investigate the role of regulatory miRNA networks related to dormancy release. The co-expression analysis grouped 759 microRNAs into two modules (MEs), module blue and module turquoise (Supplementary Figures 6A,B). Module turquoise contained 499 miRNAs (103 known miRNAs and 396 novel miRNAs) with its eigengene remained low-expressed at endodormancy stages but was strongly up-regulated after bud-burst (Supplementary Figure 6C). The rest 260 miRNAs (18 known miRNAs and 242 novel miRNAs) were clustered into module blue with the expression pattern specific to certain samples (Supplementary Figure 6D). We also carried out module-trait association analysis to identify miRNA modules correlated with bud break rate (Supplementary Figure 6B). We found module turquoise is highly correlated with BBR (*r* = 0.93, *p*-value = 1.0e^–5^). A number of known miRNAs were detected in module turquoise, such as miR156/157-x/y, miR171, miR172-y, and miR319-x/y that were differentially expressed during the process of floral bud bursting.

### Integrated miRNA-transcription factor regulatory network during dormancy release

**FIGURE 8:**
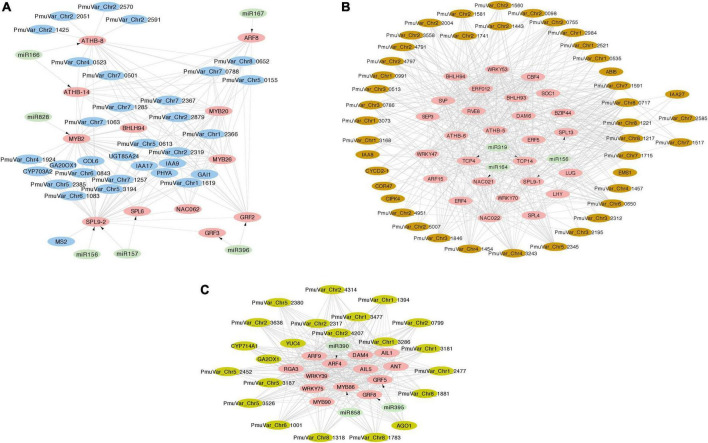
Integrated miRNA-TF regulatory networks of three co-expression gene modules. The transcription factors and target genes of DEmiRs were identified from module blue **(A)**, module brown **(B)**, and module darkseagreen4 **(C)**. The pink nodes represent hub transcription factors, the light green nodes represent miRNAs, and the other putative regulated genes were colored with their corresponding module color. The arrows represent the regulation relationship between miRNAs and their target TFs. The edges connecting TFs and other genes represent co-expression relationship.

MiRNAs function post-transcriptionally to regulate protein-coding genes through initiating cleavage or degradation (Rubio-Somoza and Weigel, 2011). Most miRNA targeted genes are transcription factors that mediate plant growth and development through regulating downstream genes (Samad et al., 2017). To identify the potential miRNA-TF-mRNA regulatory network, we first mapped all DEGs to the BBR-associated gene modules and detected 650 DEGs belonging to module blue, 894 DEGs belonging to module brown, and 164 DEGs belonging to module darkseagreen4. The differentially expressed transcription factors were extracted from these three modules along with their potential gene targets selected as the most connected genes based on the correlation matrix. Furthermore, we mapped the transcription factors targeted by DEmiRs to the co-expression network and integrated the miRNA-TF regulatory pairs (Figure 8).

In general, the co-expression analysis revealed complex miRNA-TF-mRNA networks modulating floral bud break in *P. mume*. Module blue is consisted of a few hub transcription factors, including *ATHB-8* and *ATHB-14* targeted by miR166, *GRF2* (GROWTH REGULATOR FACTOR 2) and *GRF3* targeted by miR396, *ARF8* (AUXIN RESPONSE FACTOR 8) mediated by miR167, *MYB2* targeted by miR828, and SBP transcription factors *SPL9-2* (SQUAMOSA PROMOTER-BINDING-LIKE PROTEIN 9) and *SPL6* targeted by miR156/157 (Figure 8A). Moreover, a few transcription factors related to hormonal signaling were co-expressed in module blue, such as *IAA9* (INDOLEACETIC ACID-INDUCED PROTEIN 9) and *IAA17* from AUX/IAA transcriptional regulator family, and GARS transcription factor *GAI* (GIBBERELLIC ACID INSENSITIVE) (Figure 8A). In module brown, we detected *CBF4* (C-REPEAT-BINDING FACTOR 4) and *DAM*6 (DORMANCY ASSOCIATED MADS-BOX 6), which form a regulatory module in regulating flower bud dormancy in *P. mume* (Zhao et al., 2018b; Figure 8B). Among the co-expressed transcription factors, *NAC021* targeted by miR164, *TCP4*, and *TCP14* targeted by miR319, *SPL9-1* targeted by miR156, *IAA8* and *IAA27*, and *ARF15* are putative transcription factors essential in regulating bud dormancy process in *P. mume*. The network analysis also identified a few connected genes putatively acting downstream of these transcription factors, such as UDP-Glycosyltransferase superfamily proteins (UGT71K2 and UGT87A2), and Cyclin-D2, and *CIPK4* (CBL-INTERACTING PROTEIN KINASE 4) (Figure 8B). Module darkseagreen4 also contained a few characterized bud break regulators in perennial tree species, such as *DAM4* (DORMANCY ASSOCIATED MADS-BOX 4), *ANT* (AINTEGUMENTA), and *GA2OX1* (GIBBERELLIN 2-OXIDASE 1) (Yang et al., 2021). We also identified a few miRNA-TF pairs, including miRNA390-ARF4, miR396-GRF5/GRF8, and miR585-MYB86 (Figure 8C). The co-expression network analysis provided a list of candidate genes with functions uncharacterized in floral bud break or dormancy cycling. These co-expressed genes are putative regulators or miRNA/TF target genes worthy of future examinations.

### Expression validation of differentially expressed miRNAs and their targets with quantitative real-time PCR

**FIGURE 9:**
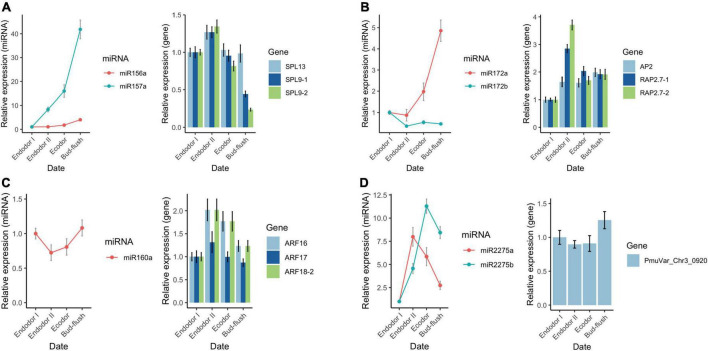
qRT-PCR analysis of the expression pattern of DEGs and DEmiRs during floral bud break in *P. mume*. The relative expression level of miRNAs within miR156/157 family **(A)**, miR172 family **(B)**, miR160 **(C)**, and miR2275 **(D)** were analyzed across four developmental stages using 5S rRNA as internal reference. Similarly, the relative expression level of their corresponding target genes were analyzed with *PP2A* as reference gene in the qRT-PCR assays.

To validate the sequencing results, we performed qRT-PCR analysis on a few differentially expressed miRNAs and their target genes. We examined the relative expression level of seven conserved miRNAs (miR156-x, miR157-x, miR160-x, miR172-x, miR172-y, miR2275-x, and miR2275-y) and their target genes across four developmental stages during floral bud dormancy release (Figure 9). As shown, the qRT-PCR results were consistent with the high-throughput sequencing data (Figure 9). miR156-x and miR157-x were gradually increased as floral bud exit dormancy, whereas their target SPL transcription factors (*SPL9-1*, *SPL9-2*, *SPL13*) were first up-regulated but then were slightly down-regulated (Figure 9A). Similarly, the expression of miR172-x constantly increased with their target genes *AP2* and *RAP2.7* transcripts displaying contrasting expression pattern as floral bud exit dormancy (Figure 9B). The expression of miR160-x was repressed during early stages of bud dormancy release, however, was significantly increased in the bud flush process. On the other hand, the ARF transcription factors including *ARF16*, *ARF17*, and *ARF18* were up-regulated during dormancy release (Figure 9C). miR2275-x and miR2275-y were first up-regulated and then down-regulated after exit endodormancy. Their predicted target gene PmuVar_Chr3_0920 was found up-regulated during bud break (Figure 9D). In general, the negative correlation between expression profiles of miRNAs and their target genes were confirmed.

## Discussion

Floral bud flush in deciduous trees is a multifaceted physiological process that involves floral bud initiation, dormancy cycling, and floral organ development (Cooke et al., 2012). In temperate fruit trees, the onset of floral bud initiation occurred in late summer with floral primordia differentiated sequentially in the order of sepals, petals, stamens, and carpels (Koutinas et al., 2014). All flower primordia continue to develop for several months before entering dormancy period in winter. In *Prunus* fruit trees, the internal floral structures of endodormant buds continued to develop during chilling accumulation (Fadón et al., 2018; Hsiang et al., 2021). After the fulfillment of chilling requirement, floral organs continue to expand and mature and finally bloom under warm conditions (Ito et al., 2016). The timing of reproductive bud break is one of the most important adaptive trait that ensures plant survival and synchronized the reproductive activities with seasonal environmental changes (Zhang et al., 2019; Fadón et al., 2020). In the context of global warming, it is crucial to understand the control of flowering phenology in deciduous tree species (Zhang et al., 2021b). Till now, the molecular mechanism underlying flower bud development and dormancy cycling is extensively explored among woody perennial species. However, the knowledge regarding to the microRNA-mediated gene regulatory networks during this process is still limited.

### Candidate genes related to floral bud dormancy transition in *Prunus mume*

In this study, we examined the morphological change of floral bud and observed floral bud enlargement accompanied by organ development and maturation. Based on the bud break competence tests, we proposed four sampling time points corresponding to key developmental stages during floral bud break. Our transcriptome analysis revealed 1,553 DEGs in endodormancy-vs.-ecodormancy comparison and 2,084 DEGs in ecodormancy-vs.-bud-burst comparison. Genes associated with endodormancy release were over-represented in biological processes, such as response to hormones (ethylene, abscisic acid, gibberellin, etc.), response to cold, and flower organ development, suggesting that the transition process from endodrmancy to ecodormancy requires complex regulatory networks coordinating together (Fan et al., 2015). Ecodormancy is a short-term period of inhibited growth and can proceed to bud break in warmer climates (Ueno et al., 2013). Genes differentially expressed between ecodormancy and bud flush stage are mostly involved in floral whorl development and gametophyte production. These results are consistent with the activated reproductive organ development at ecodormancy in apricot and peach (Yu et al., 2020).

With co-expression analysis, we identified three clusters of co-expressed genes with distinctive expression profiles highly correlated with the level of dormancy depth during floral bud break. Among the candidate genes, DAM4-6 are well-known transcription factors that are required for endodormancy maintenance in perennial trees. Previous studies have reported that the expression of *DAM4-6* is suppressed by chilling temperatures to ensure dormancy release and the expression of *DAM5* and *DAM6* was found inversely correlated with bud break rate in peach and Japanese apricot (Jiménez et al., 2010; Zhu et al., 2020). The deletion of *DAMs* in peach lead to dormancy-incapable mutants (Bielenberg et al., 2008). The silencing of *DAMs* in apple using RNA interference lead to ever-growing apple that fail to enter bud dormancy in winter (Wu et al., 2021). Moreover, we identified a few transcription factors that putatively interact with DAMs, such as CBF4 and SOC1. In *P. mume*, CBFs can regulate *DAM6* through binding its promoter or forming protein dimers with DAM6 (Zhao et al., 2018a). SOC1 protein can also interact with DAM1 and DAM5 *in vivo* and *in vitro* in *P. avium* (Wang et al., 2020). The regulatory module of *DAMs* and their putative interacting genes suggested the conserved molecular mechanism underlying dormancy control across *Prunus* tree species. In addition to DAMs, we identified SVP, another MADS-box transcription factor that is crucial to promoting bud endodormancy in poplar (Singh et al., 2018). SVL can also inhibit gibberellin biosynthetic genes, *GA20OX1* and *GA20OX2* to deepen the endodormancy state (Singh et al., 2018). In our study, we identified *ANT* (AINTEGUMENTA) along with its paralogs *AIL1* (AINTEGUMENTA-LIKE 1) and *AIL5* (AINTEGUMENTA-LIKE 5), and *CYCDs*, which are likely required for cell proliferation during floral organ development and expansion during bud break. During dormancy induction in poplar, the transcription of *ANT* is strongly inhibited, resulting in growth cessation and bud formation (Azeez et al., 2014). *ANT* is also critical for ovule initiation and formation in *Arabidopsis* (Omidbakhshfard et al., 2015). CYCD3.1 is a D-type cyclin required to promote cell division during bud break in poplar (Azeez et al., 2021).

Hormonal signaling is also essential for floral development and dormancy transition (Liu and Sherif, 2019). ABA and gibberellin are two plant hormones that are known to regulate dormancy and seed germination (Yu et al., 2013). ABI3 (ABSCISIC ACID-INSENSISTIVE 3) is a key factor acting downstream of ABA signaling. In previous studies, overexpressing ABI3 caused early bud set in poplar (Rohde et al., 2002). In our study, the expression of *ABI5* was significantly repressed, indicating that ABA deactivation is also required for dormancy release in floral buds. In early stage of chilling accumulation, the expression level of GA biosynthetic genes, such as *GA20OX1*, was relatively low in endodormant buds and then strongly up-regulated after exit endodormancy. *GA2OX1* and *GA2OX8*, involved in GA catabolic pathway, were strongly down-regulated from dormancy release to floral bud break (Figure 3). The balance between ABA and GA signaling is crucial in regulating dormancy cycling (Zhang Z. et al., 2018). Auxin is also an important plant hormone pivotal for dormancy transition in perennial tree species (Liu and Sherif, 2019). In the auxin-signaling pathway, IAA/AUX transcriptional factors can inhibit the ARFs activities through protein interactions (Leyser, 2017). We observed constantly decreasing level of *ARF9*, *ARF15*, and *IAA9*, and increasing level of *ARF8*, *ARF18*, *IAA16*, *IAA26*, and *IAA17* during the process of floral bud break. These auxin-mediated genes are likely implicated in regulating floral bud break in *P. mume*. Additionally, we detected the differential expression of a few ERF (ETHYLENE RESPONSE FACTOR) transcription factors, such as *ERF4*, *ERF5*, and *ERF113*. These ERFs are likely required in regulating abiotic stresses responses relevant to the process of floral bud break in *P. mume*.

### MicroRNAs associated with floral bud dormancy release in *Prunus mume*

Small RNAs are non-coding RNAs ranging from 20 to 24 nt and can be categorized into two major categories: small interfering RNAs (siRNA) and microRNAs (miRNA). miRNAs are usually generated from single-stranded RNA transcripts forming hairpin structure, which can be processed into mature miRNAs and fine-tune gene expression post-transcriptionally (Liu et al., 2020). Unlike miRNAs in animals, plant miRNAs mostly bind to RNA sequences within coding region of their target genes through near-perfect complementarity and direct mRNA cleavage for degradation (Millar and Waterhouse, 2005; Axtell, 2008). It has been demonstrated that the miRNA-mediated gene silencing is essential to many important biological processes including timing of development, pattern formation, response to environmental stimulus (Cui et al., 2017). With the emergence of high-throughput sequencing technology and bioinformatics tools, miRNAs and their functional roles were investigated in diverse developmental processes among a large number of plant species (Qin et al., 2014).

To identify key miRNAs involved in dormancy release in *P. mume*, we performed small RNA sequencing on floral buds and identified 41 known miRNAs and 53 novel miRNAs differentially expressed across different developmental stage comparisons. Their target genes were enriched in biological pathways, such as floral organ morphogenesis, hormonal signaling, secondary metabolism biosynthesis, and carbohydrate metabolism. Previous studies reported a few miRNAs related to dormancy cycling in perennial tree species (Ding et al., 2016). In poplar, miR169 was found to repress *HAP2* (HEME ACTIVATOR PROTEIN 2) in dormant cambium, vegetative bud and floral bud tissues (Ding et al., 2016). The transcription of *HAP2* rapidly declined as dormant bud resumes growth in the next spring (Potkar et al., 2013). In our study, miR169 maintained a constant level during dormancy release but slightly decreased after bud flush in our study. Another important miRNA identified as dormancy regulator is miR6390, which target *DAM* (DORMANCY-ASSOCIATED MADS-BOX) transcripts during floral bud dormancy release in pear (Niu et al., 2016). However, we fail to recognize miR6390 in our small RNA sequencing analysis. It is likely that there exist species-specific miRNAs that regulate dormancy release across tree species. One recent study identified a *Prunus* specific microRNA miR6285, which is differentially expressed between endodormancy and ecodormancy (Yu et al., 2021). In peach, miR6285 is a cold-responsive microRNA targeting gene *NRP* (ASPARAGINE-RICH PROTEIN) (Yu et al., 2021). We observed significantly increased level of miR6285 during dormancy release, which is consistent with its expression pattern in peach. However, we fail to validate the target gene for miR6285 with degradome sequencing, which is likely due to the low expression level of *NRP* during dormancy exit. Yu et al. also identified miR2275 that may be involved in pollen development at ecodormancy (Yu et al., 2021). We observed constantly increasing expression of miR2275 during chilling-induced dormancy release, however, miR2275 transcript level significantly dropped during the transition from ecodormancy to bud flush. In peach, miR2275 is predicted to target lncRNAs to generate phasiRNAs that is proposed to regulate microsporogenesis and pollen meiosis (Yu et al., 2021). It is likely that the functional role of miR2275 is conserved across *Prunus* species.

In addition to miRNAs characterized in previous studies, we also identified a few candidate miRNAs with expression profiles correlated with floral bud flush rate during dormancy transition. Among them, miR156/157-x/y, miR171, miR172-y, and miR319-x were identified. Previous studies have revealed that SPL transcription factors, which are target genes of miR156/157, play an important role in regulating vegetative-to-adult phase transition, pollen production, and floral organ elongation (including petals, sepals, and siliques) in *Arabidopsis* (Tsiantis et al., 2016). As plant age increases, the reduced expression of miR156 leads to increased transcript level of their target SPL transcription factors, which directly activate downstream MADS-box genes to promote flowering (Wang et al., 2009; Teotia and Tang, 2015). In the present study, miRNAs from miR156/157 family were found significantly induced with their target genes displaying contrasting expression pattern during floral bud break. The functional role of miR156/157 may be associated with floral organ expansion after the dormancy release. MiR172 is another miRNA family that controls vegetative-to-adult phase transition by targeting AP2-like transcription factors, such as AP2 in *Arabidopsis* (Aukerman and Sakai, 2003). The overexpression of miR172 triggered early flowering and disrupted floral organ by antagonizing AG (AGAMOUS) activity in *Arabidopsis* (Aukerman and Sakai, 2003). MiR172-x was found highly induced in the flushed floral bud, while *AP2* and *RAP2.7* transcripts declined after floral bud exit endodormancy. The temporal miR172-mediated down-regulation of *AP2* indicated the reduced AP2 activity in specifying floral organ identity in matured flowers (Wollmann et al., 2010). Scarecrow-like transcription factors are known targets of miR171 and were reported to regulate meristem cell maintenance, polar organization and chlorophyll synthesis (Qu et al., 2014). The overexpression of miR171 in *Arabidopsis* leads to plants with reduced shoot branching, increased chlorophyll accumulation, abnormal leaf and flower patterning (Curaba et al., 2013; Qu et al., 2014). During the transition from ecodormancy to bud flush, *SCL6* transcripts decreased as the level of miR171 dramatically increases, which may be associated with the gibberellin level change and inflorescence branching (Curaba et al., 2013). In general, we confirmed the contrasting expression pattern between many DEmiRNAs and their target genes with qRT-PCR assays. However, functional experiments are still required to clarify the regulatory mechanism of these microRNAs and their target transcription factors in controlling floral bud break in *P. mume*.

### Putative microRNA-transcription factor regulatory network controlling floral bud break

Plant microRNAs are known to target genes that are often transcription factors or genes mediating protein ubiquitination such as F-box genes (Samad et al., 2017). Ancient miRNAs play relevant functions across different plant species by targeting conserved miRNA-binding sites (Axtell, 2008). Based on sequence conservation, many bioinformatics tools were developed to search for target genes of miRNAs in new species (Chorostecki et al., 2012). In two previous small RNA studies of *P. mume*, bioinformatics approach was employed to reveal miRNA-mRNA pairs (Gao et al., 2012; Wang et al., 2013). One possible problem with pure computational prediction is high false positive rate (Naoumkina et al., 2016). Degradome sequencing is a modified 5′-RACE (Rapid Amplification of cDNA Ends) approach combined with high-throughput sequencing and has recently been used to find accurate miRNA-target relationships in many plant species (Candar-Cakir et al., 2016). In our study, we combined the computational prediction with degradome sequencing to identify miRNA-mRNA regulation relationship. We further compared the expression pattern of miRNAs and their target genes to characterize miRNA-mRNA pairs that possibly regulate dormancy release and flower opening in *P. mume*. Many target genes displayed contrasting expression pattern with their miRNAs, confirming the negative regulation between miRNAs and their targets. However, we validated only limited number of cleavage events for miRNAs, leaving out a large number of miRNAs with no target gene validated in the degradome analysis. One possible explanation could be the expression of those miRNAs was too low to cleave their target, or the sliced targets were below the level of detection in degradome sequencing (Liu et al., 2014a).

Transcription factors often activate or repress target gene expression through binding to cis-regulatory elements within target gene promoters (Maston et al., 2006). On the other hand, their expression can be regulated post-transcriptionally by miRNAs (Yu et al., 2017). To further understand the cross-talk between TFs and miRNAs, we investigated the regulatory relationship among miRNA-TFs and mRNA-mRNAs. By applying weighted co-expression network analysis (WGCNA) approach, we identified co-expressed gene modules and miRNA modules sharing similar expression patterns during dormancy release. With trait-module association analysis, we identified three gene modules (module blue, module brown, and module darkseagreen4) and one miRNA module (module turquoise) that were related to the progression of dormancy release. Co-expression network is constructed based on the pairwise correlation among genes sharing similar spatial or temporal expression profiles. The connected genes are assumed to be either directly or indirectly regulated by co-regulators (Stuart et al., 2003). Therefore, we screened the differentially expressed transcription factors as well as highly connected neighboring genes from the trait-associated modules and integrated their co-expression network with miRNA-TF regulatory pairs.

In the integrated miRNA-mRNA network, we identified a few ARFs, including *ARF8*, *ARF4*, and *ARF15* across three co-expression modules. Previous studies have reported that *ARF2-4* are targets of trans-acting siRNAs generated from miR390-targeted TAS3 gene, *ARF6* and *ARF8* are targets of miR167, and *ARF10*, *ARF16-17* are targets of miR160 in *Arabidopsis* (Axtell et al., 2006; Wu et al., 2006; Liu et al., 2010). miR167 is known to mediate stem elongation and flower organ outgrowth in *Arabidopsis* (Nagpal et al., 2005). The over-expression of miR167 lead to down-regulation of *ARF6* and *ARF8*, which further results in shortened petals, stamens, styles, and female sterility in tomato (Liu et al., 2014b). In our study, we found the expression of *ARF8* first decreased as endodormant floral buds accumulate chilling, but then strongly increased during bud flush. miR167, on the other hand, was significantly repressed during endodormancy release. The dramatic expression changes of miR167-ARF8 module suggested their possible role in floral organ growth and female fertility in *P. mume*. miR390 is implicated in regulating leaf patterning and developmental timing by repressing *ARF2*, *ARF3*, and *ARF4* (Fahlgren et al., 2006). We observed constantly increasing level of *ARF4* during flower bud flush, which indicate its activity may be required in the female and male gametophyte development in *P. mume* (Liu et al., 2018). miR396 with four targeting GRFs (*GRF2*, *GRF3*, *GRF5*, *GRF8*) were identified in module blue and module darkseagreen4. Recent findings have shown that miR396, targeting *GRF1-4* and *GRF7-9*, is essential to regulate leaf morphogenesis, root and flower development (Samad et al., 2017). In *Arabidopsis*, miR396 is important in control sepal-petal identity and pistil development by regulating *GRF* transcript levels (Liang et al., 2013). During endodormancy release, miR396 was found decreased with its targeting GRFs increasing, indicating that miR396-GRFs possibly involved in reproductive organ development in *P. mume*.

Furthermore, miR319-TCP was previously reported to function in leaf development, branching, flower development and gamete production (Fang et al., 2021). TCP transcription factors can be grouped into two classes, where class I members are mainly cell cycling activators and class II are cell cycling inhibitors. The class I transcription factors, such as *TCP14* was found involved in cell proliferation during leaf and flower development (Kieffer et al., 2011). In class II, *TCP2*, *TCP3*, and *TCP4* are important in regulating leaf development (Manassero et al., 2013). In our study, we observed constantly increasing level of *TCP4* and *TCP14* along with decreasing level of miR319-y. Another miRNA-TF pair, miR166-ATHB-8/ATHB-14 was detected in module blue. miR165/166 downregulate HD-ZIP transcription factors in regulating shoot apical meristem activity and flower development through WUSCHEL pathway (Jung and Park, 2007). It is likely these miRNA-TF pairs are involved in cell proliferation, organ polarity and vascular patterning during flower development in *P. mume*. The co-expression network analysis also detected co-expressed genes that may be regulated directly or indirectly by transcription factors. For example, we detected *ANT*, *AIL1*, and *AIL5* co-expressed with *ARFs* and *GRFs* within module darkseagreen4. In *Arabidopsis*, ARF5 upregulates the gene expression of *ANT*, *AIL1*, and *AIL6* to specify and promote flower primordia (Krizek et al., 2020). On the other hand, ANT/AIL, GRF, and ANGUSTIFOLIA3/GRF-INTERACTING FACTOR (AN3/GIF) family genes form regulatory modules to regulate cell proliferation and organ size in leaf or flower primordia (Krizek et al., 2021). Therefore, the proposed miRNA-TF mediated regulatory modules revealed potential players essential to different developmental processes occurred during endodormancy release and floral bud flush in *P. mume*.

## Conclusion

In this study, we performed comprehensive analysis using transcriptome and small RNA sequencing to reveal candidate genes and miRNAs functioning in floral bud break in *P. mume*. With differential expression analysis, we identified a number of DEGs that participated in hormonal responses, flower organ development and gametophyte production during dormancy transition. The genome-wide characterization of miRNAs identified 41 known miRNAs and 53 novel miRNAs differentially expressed during this period. Combining computational prediction tool and degradome sequencing, we identified target genes for miRNAs and validated the expression correlation between target genes and their corresponding miRNAs. With weighted co-expression analysis, we characterized the modules of genes and miRNAs displaying expression profiles that highly associated with floral bud flush competency. Based on co-expressed gene candidates, we further integrated the miRNA-TF regulatory pairs to construct the miRNA-mRNA regulatory network that possibly involved in regulating different aspects of dormancy release and floral bud break in *P. mume*. To conclude, these findings provide valuable information for understanding microRNA-mediated regulatory networks in floral bud development in *P. mume* and other woody perennials.

## Data availability statement

The datasets presented in this study can be found in online repositories. The names of the repository/repositories and accession number(s) can be found below: https://www.ncbi.nlm.nih.gov/, PRJNA833165; https://www.ncbi.nlm.nih.gov/, PRJNA832606; and https://www.ncbi.nlm.nih.gov/, PRJNA832060.

## Author contributions

MZ and QZ conceived and designed the study. MZ performed most of the experiment, analyzed the data, and wrote the manuscript. WC and XY contributed to the sample collection and qRT-PCR assays. TC and JW provided help with the data analysis. All authors contributed to the article and approved the submitted version.

## Conflict of interest

The authors declare that the research was conducted in the absence of any commercial or financial relationships that could be construed as a potential conflict of interest.

## Publisher’s note

All claims expressed in this article are solely those of the authors and do not necessarily represent those of their affiliated organizations, or those of the publisher, the editors and the reviewers. Any product that may be evaluated in this article, or claim that may be made by its manufacturer, is not guaranteed or endorsed by the publisher.

## Footnotes

1
https://CRAN.R-project.org/package=pheatmap

2
http://rna.tbi.univie.ac.at//cgi-bin/RNAWebSuite/RNAfold.cgi
