# Supplementary material for: Integrated transcriptome and small RNA sequencing in revealing miRNA-mediated regulatory network of floral bud break in Prunus mume
Source: Front Plant Sci. 2022 Jul 22;13:931454. doi: 10.3389/fpls.2022.931454 (PMC9355595; doi:10.3389/fpls.2022.931454)
Supplement: Supplementary file 1 [file Data_Sheet_1.docx]

Supplementary Material

# Supplementary Figures and Tables

Figure S1. RNA-seq analysis of floral bud samples across twelve sequencing libraries.

Figure S2. KEGG pathways enriched among DEGs identified in stage comparison endodormancy I-vs-endodormancy II, endodormancy II-vs-ecodormancy, ecodormancy-vs-bud flush.

Figure S3. The differential expression analysis identified novel miRNAs across stage comparisons.

Figure S4. Identification of the cleavage sites of miRNAs on their targets by degradome sequencing.

Figure S5. Expression pattern analysis of DEmiRs and their target genes during dormancy release in *P. mume*.

Figure S6. Co-expressed miRNA modules identified in the WGCNA analysis.

Table S1. Primer sequences for qRT-PCR analysis of selected genes and miRNAs.

Table S2. GO enrichment analysis of DEGs identified across stage comparisons.

Table S3. The association between eigen-genes of co-expressed gene modules and BBR (Bud Break Rate).

Table S4. Selected DEGs within module brown in the weighted co-expression network analysis.

Table S5. Selected DEGs within module blue in the weighted co-expression network analysis.

Table S6. Selected DEGs within module darkseagreen4 in the weighted co-expression network analysis.

Table S7. Small RNAs annotated to different categories across twelve small RNA sequencing libraries.

Table S8. Detailed information of known miRNAs identified in the small RNA sequencing analysis.

Table S9. Small RNAs annotated to different categories in the degradome sequencing.

Table S10. Top 20 GO biological process annotated to target genes of DEmiRs across stage comparison endodormancy I-vs-endodormancy II (A), endodormancy II-vs-ecodormancy (B), ecodormancy-vs-bud flush (C).

Table S11. Top 20 KEGG pathways annotated to target genes of DEmiRs across stage comparison endodormancy I-vs-endodormancy II (A), endodormancy II-vs-ecodormancy (B), ecodormancy-vs-bud flush (C).

## Supplementary Figures

Figure S1. RNA-seq analysis of floral bud samples across twelve sequencing libraries. (A) Principal component analysis showing overall gene expression profile across bud samples; (B) Heatmap showing Pearson correlation among RNA samples across twelve sequencing libraries.

Figure S2. KEGG pathways enriched among DEGs identified in stage comparison endodormancy I-vs-endodormancy II (A), endodormancy II-vs-ecodormancy (B), ecodormancy-vs-bud flush (C).

Figure S3. The differential expression analysis identified novel miRNAs across stage comparisons. A. Venn diagram of novel DEmiRs detected among stage comparisons. B. Heatmap showing hierarchical clustering structure of novel miRNAs based on expression profiles.

Figure S4. Identification of the cleavage sites of miRNAs on their targets by degradome sequencing. The X-axis indicates the mRNA position of miRNA targets from 5' to 3'. The red bar shows the degradome read with the highest count on the mRNA.

Figure S5. Expression pattern analysis of DEmiRs and their target genes during dormancy release in *P. mume*. A-B. The barplots revealing the expression levels of miR319-x/y/z (A) and their target genes (B) during floral bud break process. C-D. The barplots revealing the expression levels of miR164-x/y/z (C) and their target genes (D) during floral bud break. E-F. The barplots revealing the expression levels of miR2275-x/y/z (E) and their target genes (F) during floral bud break.


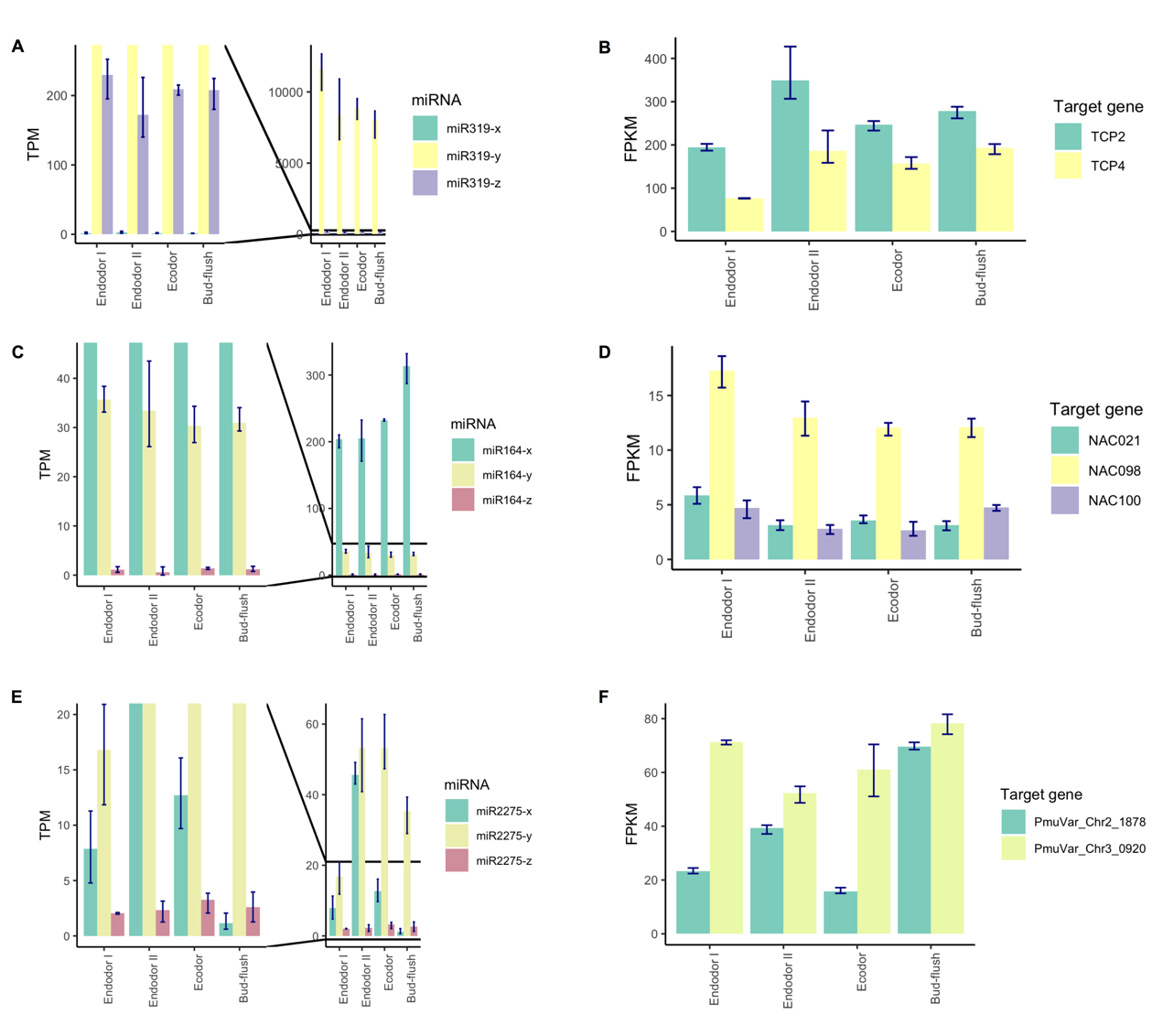


Figure S6. Co-expressed miRNA modules identified in the WGCNA analysis. (A) Clustering dendrogram of miRNAs modules. (B) miRNA module-trait association analysis. (C) barplots of eigen-miRNA in module turquoise. (D) barplots of eigen-gene in module blue.

## Supplementary Tables

Table S1. Primer sequences for qRT-PCR analysis of selected genes and miRNAs. The universal reverse primer for miRNAs were provided by miRNA SYBR Green RT-qPCR Kit.

| Gene/miRNA | Primer | Sequence |
| --- | --- | --- |
| PmuVar_Chr1_1472 | F | GTCAAGTCAACCATGGGGCT |
|  | R | AGGAAGCATCTCGTGTGAGC |
| PmuVar_Chr8_1993 | F | GTCAAATCAACCATGGGGCT |
|  | R | GGGAAGCATTTCATGTGAGC |
| PmuVar_Chr4_1953 | F | AGTATGTGGCCTGTGGAAGC |
|  | R | GCGCAAGAAAGTTTGAGGGG |
| PmuVar_Chr1_1236 | F | TTTGGGCTTGTTTGATACCG |
|  | R | TAACGCCGGATGATTCAGAG |
| PmuVar_Chr1_1333 | F | AGACTCGTCCACCCTCAACT |
|  | R | GCTCCGCAGGAGCATAGTAG |
| PmuVar_Chr5_2600 | F | TTACGGTGAGGCCAAAGACC |
|  | R | CCACCAGACTCAGCAAACGA |
| PmuVar_Chr5_2526 | F | TTCTCCGTGCCTCGTTACTG |
|  | R | GTCAAGAGGTGACGACGAGG |
| PmuVar_Chr2_0764 | F | GAGCAAGTTCGTCAACCACA |
|  | R | CCTTCCTTCCAGAACCTTCC |
| PmuVar_Chr1_3619 | F | TCTGCACATGGGATGCAAGT |
|  | R | GAGCCAAGAACCGAGAGGTC |
| PmuVar_Chr3_1567 | F | GGTATTGGAAGGCCACAGGA |
|  | R | GACCCAGTCGGATTTTCTGC |
| PmuVar_Chr3_1908 | F | ACGGCTCCACCAGCAGTATT |
|  | R | TGGTGGAGAAACAGGACACG |
| PmuVar_Chr3_0920 | F | GGATCCAGCATGGATTGGTT |
|  | R | GACCGTAGACGGAAGCCAAC |
| 5sRNA | F | GCGTAGAGGAAGCACACCAAT |
| miR156-x | F | CGTGACAGAAGAGAGTGAGCAC |
| miR157-x | F | CGTTGACAGAAGATAGAGAGCAC |
| miR160-x | F | CTGGCTCCCTGTATGCCA |
| miR172-x | F | CGGCATCATCAAGATTCACA |
| miR172-y | F | GGAATCTTGATGATGCTGCAG |
| miR2275-x | F | GAGAATTGGAGGGGACTAAACA |
| miR2275-y | F | GCTTTAGTTTCCTCCAATATCTCA |

Table S2. GO enrichment analysis of DEGs identified across stage comparisons.

| Comparison | GO term | Ontology | Description | P-value | FDR |
| --- | --- | --- | --- | --- | --- |
| Endodor I-vs-Endodor II | GO:0006950 | P | response to stress | 1.30E-24 | 2.00E-21 |
| Endodor I-vs-Endodor II | GO:0009743 | P | response to carbohydrate stimulus | 2.40E-11 | 1.10E-08 |
| Endodor I-vs-Endodor II | GO:0009266 | P | response to temperature stimulus | 1.40E-09 | 4.60E-07 |
| Endodor I-vs-Endodor II | GO:0006629 | P | lipid metabolic process | 1.40E-08 | 3.90E-06 |
| Endodor I-vs-Endodor II | GO:0009725 | P | response to hormone stimulus | 2.60E-07 | 3.40E-05 |
| Endodor I-vs-Endodor II | GO:0009416 | P | response to light stimulus | 1.90E-06 | 0.00017 |
| Endodor I-vs-Endodor II | GO:0006260 | P | DNA replication | 5.10E-06 | 0.00038 |
| Endodor I-vs-Endodor II | GO:0009737 | P | response to abscisic acid stimulus | 1.30E-05 | 0.00082 |
| Endodor I-vs-Endodor II | GO:0009408 | P | response to heat | 1.50E-05 | 0.00093 |
| Endodor I-vs-Endodor II | GO:0009409 | P | response to cold | 8.00E-05 | 0.0038 |
| Endodor I-vs-Endodor II | GO:0009753 | P | response to jasmonic acid stimulus | 0.00014 | 0.0061 |
| Endodor I-vs-Endodor II | GO:0006633 | P | fatty acid biosynthetic process | 0.00015 | 0.0064 |
| Endodor I-vs-Endodor II | GO:0015979 | P | photosynthesis | 0.00075 | 0.025 |
| Endodor I-vs-Endodor II | GO:0005975 | P | carbohydrate metabolic process | 0.0011 | 0.032 |
| Endodor I-vs-Endodor II | GO:0060918 | P | auxin transport | 0.0012 | 0.036 |
| Endodor I-vs-Endodor II | GO:0009723 | P | response to ethylene stimulus | 0.0014 | 0.038 |
| Endodor I-vs-Endodor II | GO:0009739 | P | response to gibberellin stimulus | 0.0015 | 0.04 |
| Endodor I-vs-Endodor II | GO:0009555 | P | pollen development | 0.0017 | 0.045 |
| Endodor II-vs-Ecodor | GO:0009628 | P | response to abiotic stimulus | 1.50E-12 | 9.00E-10 |
| Endodor II-vs-Ecodor | GO:0006950 | P | response to stress | 2.40E-12 | 1.30E-09 |
| Endodor II-vs-Ecodor | GO:0048856 | P | anatomical structure development | 9.80E-09 | 3.40E-06 |
| Endodor II-vs-Ecodor | GO:0015979 | P | photosynthesis | 2.60E-07 | 5.50E-05 |
| Endodor II-vs-Ecodor | GO:0016051 | P | carbohydrate biosynthetic process | 2.90E-07 | 5.70E-05 |
| Endodor II-vs-Ecodor | GO:0003006 | P | reproductive developmental process | 1.50E-06 | 0.00021 |
| Endodor II-vs-Ecodor | GO:0009266 | P | response to temperature stimulus | 4.70E-06 | 0.00055 |
| Endodor II-vs-Ecodor | GO:0048608 | P | reproductive structure development | 7.20E-06 | 0.00068 |
| Endodor II-vs-Ecodor | GO:0044042 | P | glucan metabolic process | 1.10E-05 | 0.00089 |
| Endodor II-vs-Ecodor | GO:0000003 | P | reproduction | 1.50E-05 | 0.0012 |
| Endodor II-vs-Ecodor | GO:0009409 | P | response to cold | 2.50E-05 | 0.0018 |
| Endodor II-vs-Ecodor | GO:0006260 | P | DNA replication | 3.20E-05 | 0.0022 |
| Endodor II-vs-Ecodor | GO:0048316 | P | seed development | 8.00E-05 | 0.0046 |
| Endodor II-vs-Ecodor | GO:0048646 | P | anatomical structure formation involved in morphogenesis | 0.00015 | 0.0075 |
| Endodor II-vs-Ecodor | GO:0005982 | P | starch metabolic process | 0.00027 | 0.012 |
| Endodor II-vs-Ecodor | GO:0009749 | P | response to glucose stimulus | 0.00037 | 0.015 |
| Endodor II-vs-Ecodor | GO:0009845 | P | seed germination | 0.00053 | 0.02 |
| Ecodor-vs-Budflush | GO:0009628 | P | response to abiotic stimulus | 3.60E-13 | 2.50E-10 |
| Ecodor-vs-Budflush | GO:0048856 | P | anatomical structure development | 2.40E-11 | 7.70E-09 |
| Ecodor-vs-Budflush | GO:0007049 | P | cell cycle | 2.20E-10 | 5.70E-08 |
| Ecodor-vs-Budflush | GO:0009653 | P | anatomical structure morphogenesis | 4.00E-09 | 7.90E-07 |
| Ecodor-vs-Budflush | GO:0003006 | P | reproductive developmental process | 7.00E-09 | 1.30E-06 |
| Ecodor-vs-Budflush | GO:0000003 | P | reproduction | 3.10E-08 | 5.10E-06 |
| Ecodor-vs-Budflush | GO:0048608 | P | reproductive structure development | 1.40E-07 | 1.80E-05 |
| Ecodor-vs-Budflush | GO:0006260 | P | DNA replication | 3.20E-07 | 3.40E-05 |
| Ecodor-vs-Budflush | GO:0009555 | P | pollen development | 3.20E-05 | 0.0015 |
| Ecodor-vs-Budflush | GO:0009908 | P | flower development | 9.50E-05 | 0.0038 |
| Ecodor-vs-Budflush | GO:0009725 | P | response to hormone stimulus | 0.00014 | 0.0051 |
| Ecodor-vs-Budflush | GO:0033205 | P | cytokinesis during cell cycle | 0.0003 | 0.0097 |
| Ecodor-vs-Budflush | GO:0048229 | P | gametophyte development | 0.00058 | 0.017 |
| Ecodor-vs-Budflush | GO:0009657 | P | plastid organization | 0.00077 | 0.021 |
| Ecodor-vs-Budflush | GO:0009887 | P | organ morphogenesis | 0.00092 | 0.024 |
| Ecodor-vs-Budflush | GO:0044042 | P | glucan metabolic process | 0.00095 | 0.025 |
| Ecodor-vs-Budflush | GO:0090066 | P | regulation of anatomical structure size | 0.0016 | 0.038 |

Table S3. The association between eigen-genes of co-expressed gene modules and BBR.

| Module | Budbreak_Rate.Cor | P-value |
| --- | --- | --- |
| MM.darkseagreen4 | -0.84 | 6.99E-04 |
| MM.yellow4 | -0.55 | 6.68E-02 |
| MM.brown4 | -0.38 | 2.28E-01 |
| MM.darkorange | -0.08 | 8.06E-01 |
| MM.lavenderblush3 | 0.01 | 9.82E-01 |
| MM.antiquewhite4 | 0.29 | 3.65E-01 |
| MM.lightpink4 | -0.22 | 4.89E-01 |
| MM.maroon | 0.29 | 3.69E-01 |
| MM.bisque4 | 0.11 | 7.34E-01 |
| MM.skyblue3 | -0.48 | 1.10E-01 |
| MM.blue | 0.81 | 1.56E-03 |
| MM.skyblue2 | -0.36 | 2.53E-01 |
| MM.yellowgreen | -0.17 | 5.92E-01 |
| MM.midnightblue | -0.58 | 4.90E-02 |
| MM.darkolivegreen | -0.23 | 4.80E-01 |
| MM.brown | -0.91 | 3.88E-05 |
| MM.plum2 | -0.69 | 1.38E-02 |
| MM.grey | -0.02 | 9.46E-01 |

Table S4. Selected DEGs within module brown in the weighted co-expression network analysis.

| GeneID | Module | Connectivity | Symbol | Description |
| --- | --- | --- | --- | --- |
| PmuVar_Chr1_0286 | brown | 567.016208 | ABI5 | PREDICTED: ABSCISIC ACID-INSENSITIVE 5-like protein 2 isoform X2 [Prunus mume] |
| PmuVar_Chr1_0739 | brown | 741.619842 | UFC | PREDICTED: protein UPSTREAM OF FLC-like [Prunus mume] |
| PmuVar_Chr1_0765 | brown | 610.333166 | ATHB-5 | PREDICTED: homeobox protein 5 [Prunus mume] |
| PmuVar_Chr1_0871 | brown | 966.702715 | BHLH94 | PREDICTED: transcription factor bHLH94 [Prunus mume] |
| PmuVar_Chr1_0921 | brown | 1078.16778 | SVP | PREDICTED: MADS-box protein SVP [Prunus mume] |
| PmuVar_Chr1_0971 | brown | 1185.76195 | PUB50 | PREDICTED: U-box [Prunus dulcis] |
| PmuVar_Chr1_0991 | brown | 368.096318 | DOF5.7 | PREDICTED: dof zinc finger protein DOF5.7 [Prunus mume] |
| PmuVar_Chr1_1041 | brown | 1069.16253 | HSL2 | PREDICTED: LRR receptor-like serine/threonine-protein kinase HSL2 [Prunus mume] |
| PmuVar_Chr1_1051 | brown | 870.347457 | UBC1 | PREDICTED: ubiquitin-conjugating enzyme E2 1-like [Prunus mume] |
| PmuVar_Chr1_1078 | brown | 941.573096 | SQE1 | PREDICTED: squalene epoxidase 3-like [Prunus mume] |
| PmuVar_Chr1_1288 | brown | 905.187397 | CRPK1 | Protein kinase superfamily protein [Prunus dulcis] |
| PmuVar_Chr1_1324 | brown | 589.235571 | ICR2 | interactor of constitutive active ROPs 2, chloroplastic isoform X4 [Prunus avium] |
| PmuVar_Chr1_1336 | brown | 974.350519 | RVE8 | PREDICTED: protein REVEILLE 8 [Prunus mume] |
| PmuVar_Chr1_1472 | brown | 660.292505 | SPL9-1 | PREDICTED: squamosa promoter-binding-like protein 9 [Prunus mume] |
| PmuVar_Chr1_1778 | brown | 618.5347 | PYL4 | PREDICTED: abscisic acid receptor PYL4 [Prunus mume] |
| PmuVar_Chr1_1840 | brown | 910.453628 | OFP1 | PREDICTED: transcription repressor OFP1-like [Prunus mume] |
| PmuVar_Chr1_1937 | brown | 806.837789 | -- | Late embryogenesis abundant protein [Trema orientale] |
| PmuVar_Chr1_1981 | brown | 714.854479 | RCI2A | Low temperature and salt responsive protein family [Prunus dulcis] |
| PmuVar_Chr1_1995 | brown | 645.599608 | -- | PREDICTED: MuDR family transposase [Prunus dulcis] |
| PmuVar_Chr1_2076 | brown | 859.177478 | OSH71 | PREDICTED: homeobox protein knotted-1-like 1 [Prunus mume] |
| PmuVar_Chr1_2086 | brown | 1051.8931 | CKI1 | PREDICTED: histidine kinase CKI1 [Prunus mume] |
| PmuVar_Chr1_2172 | brown | 214.437703 | CYCH1-1 | PREDICTED: cyclin-H1-1 isoform X2 [Prunus mume] |
| PmuVar_Chr1_2402 | brown | 972.025191 | FTB | PREDICTED: protein farnesyltransferase subunit beta-like [Prunus mume] |
| PmuVar_Chr1_2422 | brown | 841.257215 | LSH1 | PREDICTED: protein LIGHT-DEPENDENT SHORT HYPOCOTYLS 1 [Prunus mume] |
| PmuVar_Chr1_2466 | brown | 1128.65272 | LTL1 | PREDICTED: GDSL esterase/lipase LTL1-like [Prunus mume] |
| PmuVar_Chr1_2521 | brown | 978.24032 | ZFN1 | PREDICTED: zinc finger CCCH domain-containing protein ZFN-like isoform X3 [Prunus mume] |
| PmuVar_Chr1_2556 | brown | 750.358547 | FRS5 | PREDICTED: protein FAR1-RELATED SEQUENCE 5-like [Prunus mume] |
| PmuVar_Chr1_2561 | brown | 1134.44752 | HCT1 | PREDICTED: omega-hydroxypalmitate O-feruloyl transferase [Prunus mume] |
| PmuVar_Chr1_2967 | brown | 1183.83698 | CYP714C2 | cytochrome P450 714C2 [Prunus persica] |
| PmuVar_Chr1_3073 | brown | 834.956772 | UGT87A2 | UDP-Glycosyltransferase superfamily protein [Prunus dulcis] |
| PmuVar_Chr1_3168 | brown | 1167.37762 | UGT71K2 | PREDICTED: UDP-glycosyltransferase 71K1-like [Prunus mume] |
| PmuVar_Chr1_3700 | brown | 463.501773 | MYB36 | transcription factor RAX2-like [Prunus avium] |
| PmuVar_Chr1_4066 | brown | 441.190997 | OFP6 | PREDICTED: transcription repressor OFP6-like [Prunus mume] |
| PmuVar_Chr1_4072 | brown | 917.330697 | E2FA | transcription factor E2FB isoform X1 [Prunus persica] |
| PmuVar_Chr2_0442 | brown | 1015.3879 | IAA8 | PREDICTED: auxin-responsive protein IAA8-like isoform X2 [Prunus mume] |
| PmuVar_Chr2_0444 | brown | 1127.87243 | BHLH93 | PREDICTED: transcription factor bHLH93-like [Prunus mume] |
| PmuVar_Chr2_0454 | brown | 777.623858 | CIPK8 | PREDICTED: CBL-interacting serine/threonine-protein kinase 8 isoform X1 [Prunus mume] |
| PmuVar_Chr2_0525 | brown | 1049.14013 | DAM6 | MADS-box protein JOINTLESS-like isoform X2 [Prunus avium] |
| PmuVar_Chr2_0529 | brown | 126.217053 | -- | PREDICTED: histone-lysine N-methyltransferase [Prunus dulcis] |
| PmuVar_Chr2_0555 | brown | 1021.60889 | CYCD2-1 | PREDICTED: cyclin-D2-1-like [Prunus mume] |
| PmuVar_Chr2_0596 | brown | 703.866777 | ATHB-6 | homeobox-leucine zipper protein ATHB-6 [Prunus persica] |
| PmuVar_Chr2_0662 | brown | 1083.17642 | STP5 | PREDICTED: sugar transport protein 5 [Prunus mume] |
| PmuVar_Chr2_0755 | brown | 1083.29362 | TGA7 | PREDICTED: transcription factor TGA3 isoform X3 [Prunus mume] |
| PmuVar_Chr2_0821 | brown | 989.671288 | ERF012 | PREDICTED: ethylene-responsive transcription factor ERF012 [Prunus mume] |
| PmuVar_Chr2_0891 | brown | 232.56214 | -- | PREDICTED: F-box only 11 [Prunus dulcis] |
| PmuVar_Chr2_1002 | brown | 585.061525 | -- | histone acetyltransferase [Trema orientale] |
| PmuVar_Chr2_1059 | brown | 587.364511 | RD22 | PREDICTED: BURP domain protein RD22-like [Prunus mume] |
| PmuVar_Chr2_1064 | brown | 942.108574 | BURP3 | PREDICTED: BURP domain protein RD22-like isoform X2 [Prunus mume] |
| PmuVar_Chr2_1443 | brown | 962.167456 | ERF008 | ethylene-responsive transcription factor ERF011 [Prunus persica] |
| PmuVar_Chr2_1458 | brown | 958.550715 | CYCD3-2 | PREDICTED: cyclin-D3-1-like [Prunus mume] |
| PmuVar_Chr2_1560 | brown | 705.018326 | GRF6 | PREDICTED: growth-regulating factor 6-like [Prunus mume] |
| PmuVar_Chr2_1581 | brown | 863.509104 | KN1 | PREDICTED: homeobox protein knotted-1-like 2 [Prunus mume] |
| PmuVar_Chr2_1634 | brown | 1106.7668 | NRPD2 | PREDICTED: DNA-directed RNA polymerases IV and V subunit 2-like [Prunus mume] |
| PmuVar_Chr2_1741 | brown | 1019.77662 | MYB1 | PREDICTED: transcription factor WER-like [Prunus mume] |
| PmuVar_Chr2_2154 | brown | 1018.64425 | COR47 | PREDICTED: dehydrin COR47-like [Prunus mume] |
| PmuVar_Chr2_2753 | brown | 441.569115 | CYP734A1 | PREDICTED: cytochrome P450 CYP749A22-like [Prunus mume] |
| PmuVar_Chr2_2772 | brown | 906.048786 | CYP78A5 | cytochrome P450 78A5 [Prunus persica] |
| PmuVar_Chr2_2919 | brown | 755.015948 | TCP14 | PREDICTED: transcription factor TCP15 [Prunus mume] |
| PmuVar_Chr2_3056 | brown | 379.947987 | UGT74B1 | PREDICTED: UDP-glycosyltransferase 74B1-like [Prunus mume] |
| PmuVar_Chr2_3063 | brown | 750.795887 | CYCD1-1 | cyclin-D1-1 [Prunus persica] |
| PmuVar_Chr2_3439 | brown | 597.260398 | SEP3 | PREDICTED: agamous-like MADS-box protein AGL9 homolog [Prunus mume] |
| PmuVar_Chr2_3558 | brown | 573.158039 | ERF113 | PREDICTED: ethylene-responsive transcription factor ERF113 [Prunus mume] |
| PmuVar_Chr2_3884 | brown | 1067.87595 | CIPK4 | CBL-interacting serine/threonine-protein kinase 4 [Prunus persica] |
| PmuVar_Chr2_4617 | brown | 798.33723 | -- | PREDICTED: late embryogenesis abundant protein D-29 [Prunus mume] |
| PmuVar_Chr2_4871 | brown | 960.843204 | SUS2 | sucrose synthase 2 [Prunus persica] |
| PmuVar_Chr2_5007 | brown | 1081.24895 | ERD6 | PREDICTED: sugar transporter ERD6-like 16 [Prunus mume] |
| PmuVar_Chr3_0517 | brown | 756.626238 | ERF4 | ethylene responsive factor 9 [Prunus persica] |
| PmuVar_Chr3_0559 | brown | 693.826634 | ARF15 | PREDICTED: auxin response factor [Prunus dulcis] |
| PmuVar_Chr3_0786 | brown | 819.413986 | WRKY28 | PREDICTED: probable WRKY transcription factor 71 [Prunus mume] |
| PmuVar_Chr3_1567 | brown | 809.369365 | NAC021 | PREDICTED: NAC domain-containing protein 21/22 [Prunus mume] |
| PmuVar_Chr3_1845 | brown | 825.959193 | TCP4 | PREDICTED: transcription factor TCP4 [Prunus mume] |
| PmuVar_Chr3_1914 | brown | 1103.06934 | ERF4 | ethylene-responsive transcription factor 4 [Prunus avium] |
| PmuVar_Chr3_2136 | brown | 1092.98919 | GA2OX8 | PREDICTED: gibberellin 2-beta-dioxygenase 8 [Prunus mume] |
| PmuVar_Chr4_1454 | brown | 504.944682 | GRF5 | PREDICTED: growth-regulating factor 5 isoform X1 [Prunus mume] |
| PmuVar_Chr4_1475 | brown | 986.604736 | CYP92C6 | PREDICTED: cytochrome P450 71A1-like [Prunus mume] |
| PmuVar_Chr4_1778 | brown | 629.8982 | FCA | PREDICTED: flowering time control [Prunus dulcis] |
| PmuVar_Chr4_1846 | brown | 296.98396 | WRKY47 | PREDICTED: probable WRKY transcription factor 47 [Prunus mume] |
| PmuVar_Chr4_2466 | brown | 427.481192 | UGT83A1 | UDP-glycosyltransferase 83A1 [Prunus persica] |
| PmuVar_Chr4_2614 | brown | 1010.23128 | COL2 | PREDICTED: zinc finger protein CONSTANS-LIKE 2 [Prunus mume] |
| PmuVar_Chr4_3156 | brown | 691.179754 | UGT71A16 | PREDICTED: UDP-glycosyltransferase 71A16-like [Prunus mume] |
| PmuVar_Chr4_3243 | brown | 790.994702 | WRKY71 | PREDICTED: probable WRKY transcription factor 71 [Prunus mume] |
| PmuVar_Chr4_3405 | brown | 570.73629 | BGLU12 | PREDICTED: beta-glucosidase 12-like [Prunus mume] |
| PmuVar_Chr4_3424 | brown | 696.407957 | -- | cold-regulated protein [Trema orientale] |
| PmuVar_Chr5_2059 | brown | 872.167217 | BAM7 | beta-amylase 7-like isoform X1 [Prunus avium] |
| PmuVar_Chr5_2181 | brown | 1069.44083 | LEA14-A | PREDICTED: desiccation protectant protein Lea14 homolog [Prunus mume] |
| PmuVar_Chr5_2248 | brown | 734.260326 | WRKY53 | PREDICTED: probable WRKY transcription factor 46 [Prunus mume] |
| PmuVar_Chr5_2338 | brown | 236.055352 | SAUR32 | PREDICTED: auxin-responsive protein SAUR32 [Prunus mume] |
| PmuVar_Chr5_2345 | brown | 886.369788 | MYC2 | PREDICTED: mRNAion factor [Prunus dulcis] |
| PmuVar_Chr5_2351 | brown | 630.112165 | CIPK9 | CBL-interacting serine/threonine-protein kinase 9 isoform X1 [Prunus persica] |
| PmuVar_Chr5_2390 | brown | 754.3318 | LHY | PREDICTED: protein LHY isoform X1 [Prunus mume] |
| PmuVar_Chr5_2431 | brown | 789.957368 | NAC014 | protein NTM1-like 9 [Prunus avium] |
| PmuVar_Chr5_2750 | brown | 484.44218 | SCL9 | PREDICTED: scarecrow-like protein 14 [Prunus mume] |
| PmuVar_Chr5_2812 | brown | 629.194649 | UGT76F1 | PREDICTED: UDP-glycosyltransferase 76F1-like [Prunus mume] |
| PmuVar_Chr5_2851 | brown | 913.693495 | LAX2 | PREDICTED: auxin transporter-like protein 1 [Prunus mume] |
| PmuVar_Chr5_3012 | brown | 1061.05564 | WRKY70 | PREDICTED: probable WRKY transcription factor 70 isoform X1 [Prunus mume] |
| PmuVar_Chr5_3209 | brown | 684.849502 | GA20OX1 | PREDICTED: gibberellin 20 oxidase 1-D [Prunus mume] |
| PmuVar_Chr5_3211 | brown | 442.430994 | MADS3 | PREDICTED: MADS-box transcription factor 6-like [Prunus mume] |
| PmuVar_Chr5_3248 | brown | 518.061605 | EMS1 | PREDICTED: leucine-rich repeat receptor protein kinase EMS1 [Prunus mume] |
| PmuVar_Chr6_1115 | brown | 1069.01206 | HVA22A | PREDICTED: zinc finger protein 346-like [Prunus mume] |
| PmuVar_Chr6_1229 | brown | 1137.45759 | SUVR2 | PREDICTED: histone-lysine N-methyltransferase [Prunus dulcis] |
| PmuVar_Chr6_1659 | brown | 1037.21808 | NAC022 | PREDICTED: NAC transcription factor 29 [Prunus mume] |
| PmuVar_Chr6_2143 | brown | 674.471633 | CYP707A4 | PREDICTED: abscisic acid 8'-hydroxylase 4 [Prunus mume] |
| PmuVar_Chr6_2229 | brown | 862.847811 | NHO1 | myb family transcription factor EFM [Prunus persica] |
| PmuVar_Chr6_2430 | brown | 885.738946 | BZIP44 | PREDICTED: bZIP transcription factor 53-like [Prunus mume] |
| PmuVar_Chr7_0080 | brown | 1050.7091 | CBF4 | CBF/DREB1-like protein e-2 [Prunus mume] |
| PmuVar_Chr7_0227 | brown | 942.504899 | CYP86A22 | PREDICTED: cytochrome P450 86A22 [Prunus mume] |
| PmuVar_Chr7_0239 | brown | 417.356129 | COL4 | PREDICTED: zinc finger [Prunus dulcis] |
| PmuVar_Chr7_0535 | brown | 854.118879 | MYC4 | PREDICTED: transcription factor MYC2-like [Prunus mume] |
| PmuVar_Chr7_0652 | brown | 1067.24003 | BHLH137 | PREDICTED: transcription factor bHLH137 isoform X2 [Prunus mume] |
| PmuVar_Chr7_0688 | brown | 859.03802 | HVA22 | PREDICTED: HVA22-like protein e isoform X1 [Prunus mume] |
| PmuVar_Chr7_0699 | brown | 1115.24381 | SOC1 | PREDICTED: agamous-like MADS-box protein AGL20 [Prunus mume] |
| PmuVar_Chr7_1073 | brown | 317.773698 | HIRL1 | PREDICTED: hypersensitive-induced response protein 1 isoform X1 [Prunus mume] |
| PmuVar_Chr7_1571 | brown | 532.041479 | ZHD6 | zinc-finger homeodomain protein 11-like [Prunus avium] |
| PmuVar_Chr7_1591 | brown | 447.8841 | HDG11 | PREDICTED: homeobox-leucine zipper protein HDG11 [Prunus mume] |
| PmuVar_Chr7_1715 | brown | 322.869892 | NAC031 | PREDICTED: protein CUP-SHAPED COTYLEDON 3 [Prunus mume] |
| PmuVar_Chr7_2047 | brown | 1034.2602 | MYC2 | PREDICTED: transcription factor MYC2 [Prunus mume] |
| PmuVar_Chr7_2334 | brown | 1089.10039 | ERF5 | PREDICTED: ethylene-responsive transcription factor 5 [Prunus mume] |
| PmuVar_Chr7_2528 | brown | 461.168404 | YAB1 | PREDICTED: protein YABBY 4-like [Prunus mume] |
| PmuVar_Chr8_0539 | brown | 277.483135 | LUG | transcriptional corepressor LEUNIG isoform X2 [Prunus yedoensis var. nudiflora] [Prunus yedoensis] |
| PmuVar_Chr8_0823 | brown | 776.946619 | UGT91C1 | PREDICTED: UDP-glycosyltransferase 91C1-like [Prunus mume] |
| PmuVar_Chr8_0825 | brown | 1000.10264 | UGT91C2 | PREDICTED: UDP-glycosyltransferase 91C1-like [Prunus mume] |
| PmuVar_Chr8_1221 | brown | 622.5833 | TM6 | PREDICTED: floral homeotic protein DEFICIENS-like isoform X2 [Prunus mume] |
| PmuVar_Chr8_1615 | brown | 617.556633 | ATHB-51 | PREDICTED: homeobox-leucine zipper [Prunus dulcis] |
| PmuVar_Chr8_2268 | brown | 1025.76725 | IDM2 | PREDICTED: increased DNA methylation 2-like [Prunus mume] |
| PmuVar_Chr8_2285 | brown | 561.259563 | PTST | PREDICTED: protein PTST, chloroplastic isoform X1 [Prunus mume] |
| PmuVar_Chr8_2370 | brown | 535.1995 | NOOT1 | PREDICTED: regulatory protein NPR5 [Prunus mume] |
| PmuVar_Chr8_2749 | brown | 900.832775 | ASD1 | alpha-L-arabinofuranosidase 1 isoform X2 [Prunus persica] |

Table S5. Selected DEGs within module blue in the weighted co-expression network analysis.

| GeneID | Module | Connectivity | Symbol | Description |
| --- | --- | --- | --- | --- |
| MSTRG.1976 | blue | 656.4830249 | MYB80 | transcription factor MYB80 [Prunus persica] |
| PmuVar_Chr1_1020 | blue | 717.3684408 | IAA9 | PREDICTED: auxin-responsive protein IAA9 [Prunus mume] |
| PmuVar_Chr1_1054 | blue | 669.5124194 | HAT22 | homeobox-leucine zipper protein HAT22 [Prunus persica] |
| PmuVar_Chr1_1086 | blue | 591.7930258 | GRF2 | PREDICTED: growth-regulating factor 1 [Prunus mume] |
| PmuVar_Chr1_1088 | blue | 464.0627905 | BZIP53 | PREDICTED: light-inducible protein CPRF2 [Prunus mume] |
| PmuVar_Chr1_2366 | blue | 654.2612162 | IAA16 | PREDICTED: auxin-responsive protein IAA16 [Prunus mume] |
| PmuVar_Chr1_2804 | blue | 369.649908 | BHLH130 | PREDICTED: transcription factor bHLH130-like [Prunus mume] |
| PmuVar_Chr1_3252 | blue | 565.6809401 | UGT76A2 | PREDICTED: UDP-glucose iridoid glucosyltransferase-like [Prunus mume] |
| PmuVar_Chr1_3619 | blue | 651.772817 | ARF18 | PREDICTED: auxin response factor 18-like [Prunus mume] |
| PmuVar_Chr1_3739 | blue | 421.4988619 | CYP749A22A | PREDICTED: cytochrome P450 CYP749A22 [Prunus dulcis] |
| PmuVar_Chr1_3740 | blue | 500.3233848 | CYP749A22B | PREDICTED: cytochrome P450 CYP749A22 [Prunus dulcis] |
| PmuVar_Chr2_0089 | blue | 754.4050769 | CYP98A2 | cytochrome P450 [Striga asiatica] |
| PmuVar_Chr2_0583 | blue | 670.2020532 | BHLH96 | PREDICTED: transcription factor bHLH94-like [Prunus mume] |
| PmuVar_Chr2_0895 | blue | 595.2559789 | CYCA1-1 | PREDICTED: cyclin-A1-1-like [Prunus mume] |
| PmuVar_Chr2_1044 | blue | 819.4477591 | RD22 | BURP domain protein RD22 [Prunus persica] |
| PmuVar_Chr2_1302 | blue | 591.4970612 | ATHB-8 | PREDICTED: homeobox-leucine zipper protein ATHB-8 [Prunus mume] |
| PmuVar_Chr2_1318 | blue | 457.1956326 | IAA26 | PREDICTED: auxin-responsive protein IAA2-like [Prunus mume] |
| PmuVar_Chr2_1425 | blue | 719.2477881 | HEC2 | PREDICTED: transcription factor HEC1-like [Prunus mume] |
| PmuVar_Chr2_2384 | blue | 643.4667291 | CPSF73-II | cleavage and polyadenylation specificity factor subunit 3-II [Prunus persica] |
| PmuVar_Chr2_2570 | blue | 386.8569766 | MYB62 | PREDICTED: transcription factor MYB21 [Prunus mume] |
| PmuVar_Chr2_2591 | blue | 298.4155366 | COL16 | PREDICTED: zinc finger protein CONSTANS-LIKE 6-like [Prunus mume] |
| PmuVar_Chr2_2633 | blue | 351.9857078 | SOG1 | PREDICTED: NAC domain-containing protein 8 [Prunus mume] |
| PmuVar_Chr2_2813 | blue | 723.8105108 | BGLU40 | PREDICTED: beta-glucosidase 40-like [Prunus mume] |
| PmuVar_Chr2_2879 | blue | 820.0876206 | CYP704B1 | PREDICTED: cytochrome P450 704B1 [Prunus mume] |
| PmuVar_Chr2_2999 | blue | 820.2339263 | BHLH094 | PREDICTED: transcription factor bHLH79 [Prunus mume] |
| PmuVar_Chr2_3474 | blue | 701.9075407 | NAC083 | PREDICTED: NAC domain-containing protein 83 [Prunus mume] |
| PmuVar_Chr3_0480 | blue | 462.6142802 | NFD1 | PREDICTED: NUCLEAR FUSION DEFECTIVE 1 [Prunus mume] |
| PmuVar_Chr3_0645 | blue | 686.3588689 | -- | PREDICTED: low-temperature-induced cysteine [Prunus dulcis] |
| PmuVar_Chr3_0935 | blue | 596.1441074 | ATHB-14 | PREDICTED: homeobox-leucine zipper [Prunus dulcis] |
| PmuVar_Chr3_1194 | blue | 696.6167312 | KUP11 | PREDICTED: potassium transporter 11-like isoform X1 [Prunus mume] |
| PmuVar_Chr3_1652 | blue | 587.5106361 | CYP72A225 | PREDICTED: cytochrome P450 CYP72A219-like [Prunus mume] |
| PmuVar_Chr3_1870 | blue | 621.1639237 | UGT79B6 | PREDICTED: UDP-glycosyltransferase 79B6-like [Prunus mume] |
| PmuVar_Chr3_2025 | blue | 709.806497 | NAC056 | PREDICTED: NAC transcription factor 56 [Prunus mume] |
| PmuVar_Chr3_2692 | blue | 542.3239527 | NFYA1 | PREDICTED: nuclear transcription factor Y subunit A-1 [Prunus mume] |
| PmuVar_Chr3_2694 | blue | 454.7609097 | CPK8 | PREDICTED: calcium-dependent protein kinase 8-like [Prunus mume] |
| PmuVar_Chr4_1100 | blue | 756.5887369 | IAA17 | auxin-responsive protein IAA1 [Prunus persica] |
| PmuVar_Chr4_1400 | blue | 575.9419612 | MYB26 | PREDICTED: transcription factor MYB26 [Prunus mume] |
| PmuVar_Chr4_1695 | blue | 778.283428 | MYB20 | PREDICTED: protein ODORANT1-like [Prunus mume] |
| PmuVar_Chr4_1745 | blue | 518.047198 | ARF8 | auxin response factor 8 isoform X1 [Prunus avium] |
| PmuVar_Chr4_2329 | blue | 316.2140164 | MYB306 | PREDICTED: myb-related protein 306 [Prunus mume] |
| PmuVar_Chr4_2366 | blue | 588.1822245 | MYB16 | PREDICTED: myb-related protein 306 [Prunus mume] |
| PmuVar_Chr5_1154 | blue | 635.6722671 | NAC078 | PREDICTED: NAC domain-containing protein 78 isoform X2 [Prunus mume] |
| PmuVar_Chr5_1872 | blue | 760.3825463 | GA20OX1 | PREDICTED: gibberellin 20 oxidase 1-like [Prunus mume] |
| PmuVar_Chr5_2194 | blue | 405.1622247 | ABIL1 | PREDICTED: protein ABIL1 [Prunus mume] |
| PmuVar_Chr5_2294 | blue | 802.8368557 | CYP703A2 | PREDICTED: cytochrome P450 703A2 [Prunus mume] |
| PmuVar_Chr5_2411 | blue | 648.1023789 | NAC062 | PREDICTED: NAC domain-containing protein 45-like isoform X1 [Prunus mume] |
| PmuVar_Chr5_2818 | blue | 314.4760543 | MYB25 | PREDICTED: transcription factor RAX2-like [Prunus mume] |
| PmuVar_Chr5_2994 | blue | 707.0603836 | EXPA8 | expansin-A8 precursor [Prunus mume] |
| PmuVar_Chr5_3194 | blue | 811.6823128 | CYP704C1 | PREDICTED: cytochrome P450 704C1-like isoform X1 [Prunus mume] |
| PmuVar_Chr6_1083 | blue | 801.732448 | AUX28 | PREDICTED: auxin-induced protein AUX28-like [Prunus mume] |
| PmuVar_Chr6_1108 | blue | 540.9460922 | CYP94A2 | PREDICTED: cytochrome P450 94A1-like [Prunus mume] |
| PmuVar_Chr6_1425 | blue | 800.3033617 | CYP82G1 | PREDICTED: cytochrome P450 82G1-like [Prunus mume] |
| PmuVar_Chr6_1426 | blue | 532.1545944 | CYCC1-2 | PREDICTED: cyclin-C1-2-like [Prunus mume] |
| PmuVar_Chr6_1661 | blue | 433.665488 | LOG5 | PREDICTED: cytokinin riboside 5'-monophosphate phosphoribohydrolase LOG5 [Prunus mume] |
| PmuVar_Chr6_2751 | blue | 553.2242923 | BHLH143 | transcription factor bHLH143-like isoform X1 [Prunus yedoensis var. nudiflora] [Prunus yedoensis] |
| PmuVar_Chr7_0837 | blue | 589.317564 | CYP71AP13 | cytochrome P450 71A1-like [Prunus mume] |
| PmuVar_Chr7_1260 | blue | 543.3195432 | CYP75B2 | PREDICTED: flavonoid 3'-monooxygenase [Prunus mume] |
| PmuVar_Chr7_1285 | blue | 474.1587527 | TCP5 | PREDICTED: transcription factor TCP5 [Prunus mume] |
| PmuVar_Chr7_1307 | blue | 597.8764399 | SPL6 | teosinte glume architecture 1 isoform X1 [Prunus persica] |
| PmuVar_Chr7_1498 | blue | 446.8585612 | MYB3 | PREDICTED: myb-related protein 308-like [Prunus mume] |
| PmuVar_Chr7_1806 | blue | 563.5917474 | JMJ25 | PREDICTED: lysine-specific demethylase JMJ25-like [Prunus mume] |
| PmuVar_Chr7_2020 | blue | 661.3069603 | BHLH94 | PREDICTED: transcription factor bHLH71 [Prunus mume] |
| PmuVar_Chr7_2367 | blue | 496.9027319 | HAT4 | PREDICTED: homeobox-leucine zipper protein HAT4 [Prunus mume] |
| PmuVar_Chr7_2380 | blue | 671.293984 | MYB2 | PREDICTED: transcription factor TT2-like [Prunus mume] |
| PmuVar_Chr8_0533 | blue | 663.0407993 | GAI1 | DELLA protein [Prunus salicina] |
| PmuVar_Chr8_1579 | blue | 715.1685244 | UGT73C1 | PREDICTED: UDP-glycosyltransferase 73C1-like [Prunus mume] |
| PmuVar_Chr8_1583 | blue | 816.2805985 | UGT73C6 | PREDICTED: UDP-glycosyltransferase 73C6-like [Prunus mume] |
| PmuVar_Chr8_1664 | blue | 416.2315282 | GRF3 | PREDICTED: growth-regulating factor 3 [Prunus mume] |
| PmuVar_Chr8_1678 | blue | 793.3799208 | CYP94C1 | PREDICTED: cytochrome P450 94C1-like [Prunus mume] |
| PmuVar_Chr8_1920 | blue | 821.610834 | MS2 | MALE STERILITY 2 |
| PmuVar_Chr8_1993 | blue | 564.5156442 | SPL9-2 | PREDICTED: LOW QUALITY PROTEIN: squamosa promoter-binding-like protein 9 [Prunus mume] |

Table S6. Selected DEGs within module darkseagreen4 in the weighted co-expression network analysis.

| GeneID | Module | Connectivity | Symbol | Description |
| --- | --- | --- | --- | --- |
| PmuVar_Chr4_1452 | darkseagreen4 | 214.3943862 | AIL1 | PREDICTED: AP2-like ethylene-responsive transcription factor AIL1 [Prunus mume] |
| PmuVar_Chr2_4397 | darkseagreen4 | 35.49312661 | AIL5 | PREDICTED: AP2 [Prunus dulcis] |
| PmuVar_Chr1_1081 | darkseagreen4 | 241.7296105 | ANT | AP2-like ethylene-responsive transcription factor ANT isoform X1 [Prunus persica] |
| PmuVar_Chr7_0454 | darkseagreen4 | 255.9810832 | ARF9 | PREDICTED: auxin response factor 9 [Prunus mume] |
| PmuVar_Chr2_2503 | darkseagreen4 | 158.1212051 | ATPB | PREDICTED: ATP synthase subunit beta, mitochondrial [Prunus mume] |
| PmuVar_Chr8_1930 | darkseagreen4 | 240.4780497 | BGLU12 | beta-glucosidase 12 [Prunus persica] |
| PmuVar_Chr3_1853 | darkseagreen4 | 176.3875516 | BZIP34 | PREDICTED: basic leucine zipper 61-like isoform X1 [Prunus mume] |
| PmuVar_Chr2_1282 | darkseagreen4 | 145.6127603 | CKL4 | casein kinase I-like 3, partial [Prunus dulcis] |
| PmuVar_Chr3_0270 | darkseagreen4 | 164.9463251 | CRK29 | cysteine-rich receptor-like protein kinase 25 [Prunus persica] |
| PmuVar_Chr5_3035 | darkseagreen4 | 176.8643999 | CYP714A1 | PREDICTED: cytochrome P450 714A1-like [Prunus mume] |
| PmuVar_Chr1_2968 | darkseagreen4 | 233.1717576 | CYP714C2 | PREDICTED: cytochrome P450 714C2-like [Prunus mume] |
| PmuVar_Chr2_3638 | darkseagreen4 | 224.2733444 | CYP716A15 | PREDICTED: beta-amyrin 28-oxidase-like [Prunus mume] |
| PmuVar_Chr2_4207 | darkseagreen4 | 104.3842377 | CYP73A12 | PREDICTED: cytochrome P450 CYP73A100-like [Prunus mume] |
| PmuVar_Chr1_3181 | darkseagreen4 | 254.1845826 | CYP86A1 | PREDICTED: cytochrome P450 86A1 [Prunus mume] |
| PmuVar_Chr2_0090 | darkseagreen4 | 223.1925368 | CYP98A2 | cytochrome P450 98A2 [Prunus persica] |
| PmuVar_Chr2_0526 | darkseagreen4 | 227.0653895 | DAM4 | MADS-box protein JOINTLESS isoform X1 [Prunus persica] |
| PmuVar_Chr3_3383 | darkseagreen4 | 58.69909833 | DGS1 | PREDICTED: protein DGS1, mitochondrial [Prunus mume] |
| PmuVar_Chr3_2433 | darkseagreen4 | 79.68134833 | FRL1 | PREDICTED: FRIGIDA-like protein 1 isoform X2 [Prunus mume] |
| PmuVar_Chr3_0841 | darkseagreen4 | 218.2364535 | GA2OX1 | PREDICTED: gibberellin 2-beta-dioxygenase-like [Prunus mume] |
| PmuVar_Chr3_1406 | darkseagreen4 | 200.4725423 | GAPCP1 | PREDICTED: glyceraldehyde-3-phosphate dehydrogenase GAPCP1, chloroplastic [Prunus mume] |
| PmuVar_Chr8_2304 | darkseagreen4 | 215.4488722 | GNS1 | PREDICTED: glucan endo-1,3-beta-glucosidase, basic isoform-like [Prunus mume] |
| PmuVar_Chr7_1883 | darkseagreen4 | 226.1128619 | GRF3 | PREDICTED: growth-regulating factor 4-like [Prunus mume] |
| PmuVar_Chr7_0543 | darkseagreen4 | 204.1315427 | GRF8 | growth-regulating factor 8 isoform X1 [Prunus persica] |
| PmuVar_Chr5_2565 | darkseagreen4 | 204.8055976 | GTE6 | PREDICTED: transcription factor GTE6 [Prunus mume] |
| PmuVar_Chr6_2661 | darkseagreen4 | 221.9103703 | HVA22A | PREDICTED: HVA22 [Prunus dulcis] |
| PmuVar_Chr2_0799 | darkseagreen4 | 231.961242 | LAX3 | auxin transporter-like protein 3 [Prunus avium] |
| PmuVar_Chr2_1513 | darkseagreen4 | 191.177823 | LBD39 | PREDICTED: LOB domain-containing protein 37-like [Prunus mume] |
| PmuVar_Chr2_4479 | darkseagreen4 | 222.6031295 | MDL2 | PREDICTED: (R)-mandelonitrile lyase 1-like [Prunus mume] |
| PmuVar_Chr1_2477 | darkseagreen4 | 218.2491562 | MYB113 | PREDICTED: transcription factor MYB90-like [Prunus mume] |
| PmuVar_Chr7_0890 | darkseagreen4 | 247.2094895 | MYB86 | PREDICTED: transcription factor MYB86-like [Prunus mume] |
| PmuVar_Chr4_3370 | darkseagreen4 | 200.499637 | MYB90 | Myb10 V1-1/V2 [Prunus avium] |
| PmuVar_Chr7_2060 | darkseagreen4 | 107.4047009 | PYL1 | PREDICTED: abscisic acid receptor PYR1 isoform X1 [Prunus mume] |
| PmuVar_Chr4_1029 | darkseagreen4 | 204.4732841 | RGA3 | disease resistance protein RGA2-like [Rosa chinensis] |
| PmuVar_Chr8_0943 | darkseagreen4 | 223.8023867 | SS | PREDICTED: sucrose synthase [Prunus mume] |
| PmuVar_Chr5_1358 | darkseagreen4 | 114.9787805 | TFL2 | PREDICTED: TERMINAL FLOWER 2 [Prunus mume] |
| PmuVar_Chr8_2312 | darkseagreen4 | 214.2165388 | TL1 | PREDICTED: glucan endo-1,3-beta-glucosidase [Prunus mume] |
| PmuVar_Chr2_0781 | darkseagreen4 | 210.0230885 | UGT74E2 | PREDICTED: UDP-glycosyltransferase 74E2-like [Prunus mume] |
| PmuVar_Chr6_1882 | darkseagreen4 | 168.9311022 | UGT74G1 | PREDICTED: UDP-glycosyltransferase 74G1-like [Prunus mume] |
| PmuVar_Chr1_2378 | darkseagreen4 | 210.472861 | WRKY39 | PREDICTED: probable WRKY transcription factor 74 [Prunus mume] |
| PmuVar_Chr4_1952 | darkseagreen4 | 147.8977266 | WRKY75 | PREDICTED: probable WRKY mRNAion factor [Prunus dulcis] |
| PmuVar_Chr2_1125 | darkseagreen4 | 248.7835769 | YUC4 | PREDICTED: probable indole-3-pyruvate monooxygenase YUCCA4 [Prunus mume] |

Table S7. Small RNAs annotated to different categories across twelve small RNA sequencing libraries.

| Sample | Total | rRNA | snRNA | snoRNA | tRNA | Exon sense | Known miRNA | Novel miRNA | Other_genome_regions | Unannotated |
| --- | --- | --- | --- | --- | --- | --- | --- | --- | --- | --- |
| Endodor I_1 | 13654893 | 2552054 (18.69%) | 11859 (0.09%) | 33681 (0.25%) | 42821 (0.31%) | 520176 (3.81%) | 4193867 (30.71%) | 61015 (0.45%) | 3543432 (25.95%) | 2695988 (19.74%) |
| Endodor I_2 | 12170055 | 2800553 (23.01%) | 13879 (0.11%) | 36740 (0.30%) | 40169 (0.33%) | 439476 (3.61%) | 3515993 (28.89%) | 54096 (0.44%) | 2987137 (24.54%) | 2282012 (18.75%) |
| Endodor I_3 | 15510770 | 2195683 (14.16%) | 11177 (0.07%) | 25634 (0.17%) | 56768 (0.37%) | 573735 (3.70%) | 5158346 (33.26%) | 75988 (0.49%) | 4273746 (27.55%) | 3139693 (20.24%) |
| Endodor II_1 | 13295102 | 2589824 (19.48%) | 18293 (0.14%) | 37656 (0.28%) | 70263 (0.53%) | 517673 (3.89%) | 4164209 (31.32%) | 68166 (0.51%) | 3398533 (25.56%) | 2430485 (18.28%) |
| Endodor II_2 | 15657088 | 3041814 (19.43%) | 18164 (0.12%) | 46223 (0.30%) | 86149 (0.55%) | 605535 (3.87%) | 5012696 (32.02%) | 77909 (0.50%) | 3908057 (24.96%) | 2860541 (18.27%) |
| Endodor II_3 | 17509413 | 3348276 (19.12%) | 22958 (0.13%) | 50065 (0.29%) | 101285 (0.58%) | 758136 (4.33%) | 4683363 (26.75%) | 97601 (0.56%) | 4991979 (28.51%) | 3455750 (19.74%) |
| Ecodor_1 | 17799771 | 2044967 (11.49%) | 14671 (0.08%) | 37064 (0.21%) | 91184 (0.51%) | 874809 (4.91%) | 5357740 (30.10%) | 107673 (0.60%) | 5390603 (30.28%) | 3881060 (21.80%) |
| Ecodor_2 | 14908675 | 1754191 (11.77%) | 12724 (0.09%) | 33018 (0.22%) | 87513 (0.59%) | 731748 (4.91%) | 4322976 (29.00%) | 93555 (0.63%) | 4560338 (30.59%) | 3312612 (22.22%) |
| Ecodor_3 | 13691857 | 1536492 (11.22%) | 10890 (0.08%) | 26824 (0.20%) | 64051 (0.47%) | 677926 (4.95%) | 4289995 (31.33%) | 80684 (0.59%) | 4114006 (30.05%) | 2890989 (21.11%) |
| BFlush_1 | 15100088 | 3295190 (21.82%) | 19040 (0.13%) | 37532 (0.25%) | 109092 (0.72%) | 757326 (5.02%) | 3197453 (21.18%) | 83160 (0.55%) | 4361672 (28.89%) | 3239623 (21.45%) |
| BFlush_2 | 14602179 | 1817193 (12.44%) | 12716 (0.09%) | 31075 (0.21%) | 107659 (0.74%) | 764639 (5.24%) | 3804896 (26.06%) | 86124 (0.59%) | 4582172 (31.38%) | 3395705 (23.25%) |
| BFlush_3 | 14694467 | 2283753 (15.54%) | 15024 (0.10%) | 37444 (0.25%) | 123351 (0.84%) | 771813 (5.25%) | 3884260 (26.43%) | 82497 (0.56%) | 4345091 (29.57%) | 3151234 (21.45%) |

Table S8. Detailed information of known miRNAs identified in the small RNA sequencing analysis.

| miRNA_id | Sequence | Length | Mature_arm | Chromosome | Hairpin_start | Hairpin_end | Hairpin_strand | Hairpin_length | Hairpin_energe (kcal/mol) | Hairpin_GC | Hairpin_MFEI |
| --- | --- | --- | --- | --- | --- | --- | --- | --- | --- | --- | --- |
| miR164-x | TGGAGAAGCAGGGCACGTGCA | 21 | 5p | Chr1 | 3342244 | 3342382 | + | 139 | -65.9 | 50.36 | 0.94 |
| miR164-y | CACGTGCTCCCCTTCTCCAAC | 21 | 3p | Chr1 | 3342244 | 3342382 | + | 139 | -65.9 | 50.36 | 0.94 |
| miR8129-x | TATCCGCACATTATTATCTTG | 21 | 5p | Chr1 | 10661252 | 10661378 | + | 127 | -44.64 | 43.31 | 0.81 |
| miR390-x | AAGCTCAGGAGGGATAGCGCC | 21 | 5p | Chr1 | 15582403 | 15582514 | + | 112 | -53.3 | 44.64 | 1.07 |
| miR390-y | CGCTATCCATCCTGAGTTTCA | 21 | 3p | Chr1 | 15582403 | 15582514 | + | 112 | -53.3 | 44.64 | 1.07 |
| miR164-x | TGGAGAAGCAGGGCACGTGCA | 21 | 5p | Chr1 | 15739584 | 15739763 | + | 180 | -58.9 | 40.56 | 0.81 |
| miR6295-z | GAGGACAGAAGATGATTCAGC | 21 | 3p | Chr1 | 16752527 | 16752818 | + | 292 | -94.7 | 37.33 | 0.87 |
| miR164-x | TGGAGAAGCAGGGCACGTGCA | 21 | 5p | Chr1 | 17730124 | 17730263 | + | 140 | -71.4 | 48.57 | 1.05 |
| miR164-y | CACGTGCTCCCCTTCTCCAAC | 21 | 3p | Chr1 | 17730124 | 17730263 | + | 140 | -71.4 | 48.57 | 1.05 |
| miR167-x | TGAAGCTGCCAGCATGATCTGA | 22 | 5p | Chr1 | 18877461 | 18877545 | + | 85 | -39.9 | 40 | 1.17 |
| miR167-z | TGAAGCTGCCAGCATGATCTGAGC | 24 | 5p | Chr1 | 18877461 | 18877545 | + | 85 | -39.9 | 40 | 1.17 |
| miR481-z | TAGGACCTCACTTAAGGGCT | 20 | 5p | Chr1 | 18895836 | 18896062 | + | 227 | -132.1 | 41.41 | 1.41 |
| miR6266-z | TAAATGCAGGGGCAAAATGAT | 21 | 5p | Chr1 | 22415197 | 22415423 | + | 227 | -55.2 | 19.82 | 1.23 |
| miR6266-z | TAAATGCAGGGGCAAAATGAT | 21 | 5p | Chr1 | 25963095 | 25963351 | + | 257 | -61 | 21.01 | 1.13 |
| miR391-x | TACGCAGGAGAGATGGCGCCGT | 22 | 5p | Chr1 | 27200159 | 27200257 | + | 99 | -54.9 | 60.61 | 0.92 |
| miR8126-x | TTCTGAGTCAGATTACTGAAT | 21 | 5p | Chr1 | 29140725 | 29140850 | + | 126 | -49.8 | 38.1 | 1.04 |
| miR8126-y | TTCAGTGTTTTGACTCAGAAA | 21 | 3p | Chr1 | 29140725 | 29140850 | + | 126 | -49.8 | 38.1 | 1.04 |
| miR7782-y | AACCCGCTCTGATACCATGTT | 21 | 5p | Chr1 | 5612457 | 5612772 | - | 316 | -66.5 | 37.66 | 0.56 |
| miR5248-z | CTTTTCAGTTGGCATGCCTTC | 21 | 5p | Chr1 | 7018866 | 7019061 | - | 196 | -55 | 43.37 | 0.65 |
| miR6274-z | TTGCTATCTCCGGCCAATAAC | 21 | 5p | Chr1 | 7555076 | 7555176 | - | 101 | -42.6 | 33.66 | 1.25 |
| miR2111-x | TAATCTGCATCCTGAGGTTTA | 21 | 5p | Chr1 | 14078944 | 14079052 | - | 109 | -53.6 | 48.62 | 1.01 |
| miR2111-y | GTCCTTGGGATGCGGATTACC | 21 | 3p | Chr1 | 14078944 | 14079052 | - | 109 | -53.6 | 48.62 | 1.01 |
| miR6277-z | TGTGTGTGGAAAGAGCGAGAC | 21 | 3p | Chr1 | 19466649 | 19466746 | - | 98 | -59.2 | 42.86 | 1.41 |
| miR319-y | TTGGACTGAAGGGAGCTCCCT | 21 | 3p | Chr1 | 23193841 | 23194035 | - | 195 | -82.9 | 43.08 | 0.99 |
| miR390-x | AAGCTCAGGAGGGATAGCGCC | 21 | 5p | Chr1 | 23753912 | 23754036 | - | 125 | -56.6 | 40 | 1.13 |
| miR390-y | CGCTATCCATCCTGAGTTTCA | 21 | 3p | Chr1 | 23753912 | 23754036 | - | 125 | -56.6 | 40 | 1.13 |
| miR172-x | GCGGCATCATCAAGATTCACA | 21 | 5p | Chr1 | 25245273 | 25245455 | - | 183 | -78.8 | 41.53 | 1.04 |
| miR6291-x | ACCACATTTGTAGATCACCTT | 21 | 5p | Chr1 | 25355238 | 25355351 | - | 114 | -27.8 | 30.7 | 0.79 |
| miR391-x | TACGCAGGAGAGATGGCGCCGT | 22 | 5p | Chr1 | 26965819 | 26965916 | - | 98 | -51.9 | 60.2 | 0.88 |
| miR394-x | TTGGCATTCTGTCCACCTCC | 20 | 5p | Chr2 | 1843298 | 1843412 | + | 115 | -47.5 | 41.74 | 0.99 |
| miR8129-y | TAATAATGTCCGGATGTCCGC | 21 | 3p | Chr2 | 10265101 | 10265234 | + | 134 | -47.6 | 37.31 | 0.95 |
| miR394-x | TTGGCATTCTGTCCACCTCC | 20 | 5p | Chr2 | 15189780 | 15189880 | + | 101 | -43.4 | 44.55 | 0.96 |
| miR6288-z | AGAAAATGACAAGTGACTAGT | 21 | 3p | Chr2 | 15419249 | 15419369 | + | 121 | -51.2 | 29.75 | 1.42 |
| miR6266-z | TAAATGCAGGGGCAAAATGAT | 21 | 5p | Chr2 | 19198302 | 19198555 | + | 254 | -56.3 | 23.62 | 0.94 |
| miR398-x | GGAGTGATGCTGAGAACACAAG | 22 | 5p | Chr2 | 19815240 | 19815357 | + | 118 | -46.8 | 38.98 | 1.02 |
| miR398-y | TTGTGTTCTCAGGTCACCCCT | 21 | 3p | Chr2 | 19815240 | 19815357 | + | 118 | -46.8 | 38.98 | 1.02 |
| miR395-x | GTTCCCTTGACCACTTCATTG | 21 | 5p | Chr2 | 20517870 | 20517964 | + | 95 | -45.9 | 41.05 | 1.18 |
| miR395-x | GTTCCCTTGACCACTTCATTG | 21 | 5p | Chr2 | 20535958 | 20536231 | + | 274 | -101.7 | 41.61 | 0.89 |
| miR395-x | GTTCCCTTGACCACTTCATTG | 21 | 3p | Chr2 | 20536187 | 20536342 | + | 156 | -59.5 | 39.1 | 0.98 |
| miR6258-z | TTCCAGCTGTAAAGATCAAGA | 21 | 5p | Chr2 | 25506146 | 25506319 | + | 174 | -90.8 | 45.98 | 1.14 |
| miR8133-x | TCCTTTGCGGACGTCCAGAAG | 21 | 5p | Chr2 | 25797133 | 25797277 | + | 145 | -45.57 | 46.9 | 0.67 |
| miR7122-x | TTATACAATGAAATCACGGCCG | 22 | 5p | Chr2 | 27766133 | 27766261 | + | 129 | -49.7 | 38.76 | 0.99 |
| miR7122-y | CCGTGTTTCTTTGTATAAAG | 20 | 3p | Chr2 | 27766133 | 27766261 | + | 129 | -49.7 | 38.76 | 0.99 |
| miR7122-y | CCGTGTTTCTTTGTATAAAG | 20 | 3p | Chr2 | 27843042 | 27843157 | + | 116 | -35 | 37.93 | 0.8 |
| miR169-z | CAAGGATGACTTGCCGGCATT | 21 | 5p | Chr2 | 31137188 | 31137279 | + | 92 | -44.8 | 46.74 | 1.04 |
| miR169-z | CAAGGATGACTTGCCGGCATT | 21 | 5p | Chr2 | 31183599 | 31183690 | + | 92 | -46.7 | 47.83 | 1.06 |
| miR403-y | TTAGATTCACGCACAAACTCG | 21 | 3p | Chr2 | 34685504 | 34685620 | + | 117 | -46 | 47.01 | 0.84 |
| miR4414-x | AGCTGCTGACTCGTTGGTTCA | 21 | 5p | Chr2 | 17509709 | 17509833 | - | 125 | -50.4 | 48.8 | 0.83 |
| miR482-z | TCTTTCCTACTCCACCCATTCC | 22 | 3p | Chr2 | 17698732 | 17698831 | - | 100 | -47.5 | 44 | 1.08 |
| miR482-y | CTTCCCAAACCTCCCATTCCTA | 22 | 3p | Chr2 | 17703195 | 17703313 | - | 119 | -46.4 | 42.02 | 0.93 |
| miR2118-z | CTACCGATTCCACCCATTCCGA | 22 | 3p | Chr2 | 17707001 | 17707100 | - | 100 | -43.4 | 43 | 1.01 |
| miR395-x | GTTCCCTTGACCACTTCATTG | 21 | 5p | Chr2 | 20550667 | 20550785 | - | 119 | -44.7 | 42.86 | 0.88 |
| miR166-y | TCGGACCAGGCTTCATTCCCC | 21 | 3p | Chr2 | 27737327 | 27737430 | - | 104 | -49.4 | 46.15 | 1.03 |
| miR7782-y | AACCCGCTCTGATACCATGTT | 21 | 3p | Chr2 | 31215396 | 31215535 | - | 140 | -23.1 | 39.29 | 0.42 |
| miR6266-z | TAAATGCAGGGGCAAAATGAT | 21 | 5p | Chr2 | 43731478 | 43731745 | - | 268 | -54.19 | 22.01 | 0.92 |
| miR6266-z | TAAATGCAGGGGCAAAATGAT | 21 | 5p | Chr2 | 44622702 | 44622985 | - | 284 | -62.6 | 22.18 | 0.99 |
| miR397-x | ATTGAGTGCAGCGTTGATGAA | 21 | 5p | Chr3 | 1906479 | 1906558 | + | 80 | -42.2 | 37.5 | 1.41 |
| miR397-z | TTGAGTGCAGCGTTGATGAAT | 21 | 5p | Chr3 | 1906479 | 1906558 | + | 80 | -42.2 | 37.5 | 1.41 |
| miR397-y | TCAACGCTGCACTCAATGATG | 21 | 3p | Chr3 | 2067752 | 2067842 | + | 91 | -39.9 | 36.26 | 1.21 |
| miR397-y | TCAACGCTGCACTCAATGATG | 21 | 3p | Chr3 | 2068806 | 2068893 | + | 88 | -32.6 | 35.23 | 1.05 |
| miR399-y | TGCCAAAGGAGAATTGCCCTG | 21 | 3p | Chr3 | 4057935 | 4058050 | + | 116 | -42.4 | 44.83 | 0.82 |
| miR399-y | TGCCAAAGGAGAATTGCCCTG | 21 | 3p | Chr3 | 4090354 | 4090469 | + | 116 | -37.6 | 43.97 | 0.74 |
| miR399-y | TGCCAAAGGAGAATTGCCCTG | 21 | 3p | Chr3 | 4111983 | 4112098 | + | 116 | -32.8 | 42.24 | 0.67 |
| miR399-y | TGCCAAAGGAGAATTGCCCTG | 21 | 3p | Chr3 | 4117527 | 4117653 | + | 127 | -56.4 | 40.16 | 1.11 |
| miR160-x | TGCCTGGCTCCCTGTATGCCA | 21 | 5p | Chr3 | 6888964 | 6889065 | + | 102 | -62.9 | 53.92 | 1.14 |
| miR8129-y | TAATAATGTCCGGATGTCCGC | 21 | 3p | Chr3 | 15617202 | 15617335 | + | 134 | -47.57 | 41.79 | 0.85 |
| miR6294-z | TGGTGTAGGCTAATCACAATC | 21 | 3p | Chr3 | 20434562 | 20434777 | + | 216 | -107.5 | 37.5 | 1.33 |
| miR391-z | TACGCAGGAGAGATGGCACCG | 21 | 5p | Chr3 | 25716582 | 25716679 | + | 98 | -56.2 | 58.16 | 0.99 |
| miR397-y | TCAACGCTGCACTCAATGATG | 21 | 3p | Chr3 | 1906475 | 1906565 | - | 91 | -39.9 | 36.26 | 1.21 |
| miR397-x | ATTGAGTGCAGCGTTGATGAA | 21 | 5p | Chr3 | 2067759 | 2067838 | - | 80 | -42.2 | 37.5 | 1.41 |
| miR397-z | TTGAGTGCAGCGTTGATGAAT | 21 | 5p | Chr3 | 2067759 | 2067838 | - | 80 | -42.2 | 37.5 | 1.41 |
| miR397-x | ATTGAGTGCAGCGTTGATGAA | 21 | 5p | Chr3 | 2068810 | 2068889 | - | 80 | -36.4 | 36.25 | 1.26 |
| miR397-z | TTGAGTGCAGCGTTGATGAAT | 21 | 5p | Chr3 | 2068810 | 2068889 | - | 80 | -36.4 | 36.25 | 1.26 |
| miR2111-x | TAATCTGCATCCTGAGGTTTA | 21 | 5p | Chr3 | 6701378 | 6701644 | - | 267 | -110.8 | 37.08 | 1.12 |
| miR398-z | CGTGTTCTCAGGTCGCCCCTG | 21 | 3p | Chr3 | 23663041 | 23663165 | - | 125 | -74.9 | 57.6 | 1.04 |
| miR6270-z | TTCTGGTATTGGAATTTCATT | 21 | 3p | Chr3 | 25040661 | 25041022 | - | 362 | -107.1 | 37.02 | 0.8 |
| miR477-x | TCCCTCAAGGGCTCCCAATATT | 22 | 5p | Chr4 | 1666310 | 1666430 | + | 121 | -59.6 | 47.93 | 1.03 |
| miR477-y | GTTGGGGGCTCTTTTGGGACG | 21 | 3p | Chr4 | 1666310 | 1666430 | + | 121 | -59.6 | 47.93 | 1.03 |
| miR477-x | TCCCTCAAGGGCTCCCAATATT | 22 | 5p | Chr4 | 1670990 | 1671111 | + | 122 | -57.3 | 46.72 | 1.01 |
| miR477-y | GTTGGGGGCTCTTTTGGGACG | 21 | 3p | Chr4 | 1670990 | 1671111 | + | 122 | -57.3 | 46.72 | 1.01 |
| miR6289-z | TCCTTTGAATGGTTAGGCTCA | 21 | 3p | Chr4 | 11108615 | 11108719 | + | 105 | -52.7 | 40 | 1.25 |
| miR157-x | TTGACAGAAGATAGAGAGCAC | 21 | 5p | Chr4 | 17694259 | 17694382 | + | 124 | -52.4 | 37.1 | 1.14 |
| miR3627-z | TTCCATCTTCCTGTGACATGA | 21 | 3p | Chr4 | 18812657 | 18812756 | + | 100 | -55.7 | 50 | 1.11 |
| miR3627-x | TCGCAGGAGAGATGGCACTGTC | 22 | 5p | Chr4 | 18812884 | 18812992 | + | 109 | -56.3 | 45.87 | 1.13 |
| miR3627-y | CTGGTGTCATCCCTCCTGTGACC | 23 | 3p | Chr4 | 18812884 | 18812992 | + | 109 | -56.3 | 45.87 | 1.13 |
| miR5225-x | TCTGTCGTAGGAGAGATGGAGC | 22 | 5p | Chr4 | 18815139 | 18815290 | + | 152 | -65.4 | 44.08 | 0.98 |
| miR5225-y | TCATCTCTCCTCGACTGAAG | 20 | 3p | Chr4 | 18815139 | 18815290 | + | 152 | -65.4 | 44.08 | 0.98 |
| miR157-x | TTGACAGAAGATAGAGAGCAC | 21 | 5p | Chr4 | 909013 | 909147 | - | 135 | -50.7 | 47.41 | 0.79 |
| miR157-y | GCTCTCTATGCTTCTGTCATC | 21 | 3p | Chr4 | 909013 | 909147 | - | 135 | -50.7 | 47.41 | 0.79 |
| miR157-x | TTGACAGAAGATAGAGAGCAC | 21 | 5p | Chr4 | 909205 | 909339 | - | 135 | -52.1 | 42.96 | 0.9 |
| miR157-y | GCTCTCTATGCTTCTGTCATC | 21 | 3p | Chr4 | 909205 | 909339 | - | 135 | -52.1 | 42.96 | 0.9 |
| miR156-z | CTGACAGAAGATAGAGAGCAC | 21 | 5p | Chr4 | 909786 | 909910 | - | 125 | -55 | 45.6 | 0.96 |
| miR157-y | GCTCTCTATGCTTCTGTCATC | 21 | 3p | Chr4 | 909786 | 909910 | - | 125 | -55 | 45.6 | 0.96 |
| miR482-x | GGAATGGGCTGTTTGGGATGA | 21 | 5p | Chr4 | 936199 | 936298 | - | 100 | -49.5 | 42 | 1.18 |
| miR6266-z | TAAATGCAGGGGCAAAATGAT | 21 | 5p | Chr4 | 5900674 | 5900957 | - | 284 | -67 | 22.89 | 1.03 |
| miR1511-y | ACCTGGCTCTGATACCATAAC | 21 | 3p | Chr4 | 7361502 | 7361605 | - | 104 | -32.9 | 40.38 | 0.78 |
| miR6266-z | TAAATGCAGGGGCAAAATGAT | 21 | 5p | Chr4 | 11973177 | 11973336 | - | 160 | -33.81 | 21.88 | 0.97 |
| miR5248-z | CTTTTCAGTTGGCATGCCTTC | 21 | 5p | Chr4 | 15834274 | 15834460 | - | 187 | -55.1 | 44.39 | 0.66 |
| miR408-z | ACAGGGAACAGGTAGAGCATG | 21 | 5p | Chr4 | 16243879 | 16244005 | - | 127 | -50.8 | 48.82 | 0.82 |
| miR408-y | TGCACTGCCTCTTCCCTGGCT | 21 | 3p | Chr4 | 16243879 | 16244005 | - | 127 | -50.8 | 48.82 | 0.82 |
| miR6274-z | TTGCTATCTCCGGCCAATAAC | 21 | 5p | Chr4 | 16974264 | 16974364 | - | 101 | -23.2 | 33.66 | 0.68 |
| miR2275-x | AGAATTGGAGGGGACTAAACA | 21 | 5p | Chr4 | 19045809 | 19045931 | - | 123 | -52.5 | 34.15 | 1.25 |
| miR2275-y | TTTAGTTTCCTCCAATATCTCA | 22 | 3p | Chr4 | 19045809 | 19045931 | - | 123 | -52.5 | 34.15 | 1.25 |
| miR2275-y | TTTAGTTTCCTCCAATATCTCA | 22 | 5p | Chr4 | 19048839 | 19048959 | - | 121 | -34.3 | 31.4 | 0.9 |
| miR6274-z | TTGCTATCTCCGGCCAATAAC | 21 | 5p | Chr4 | 19452481 | 19452582 | - | 102 | -40.9 | 30.39 | 1.32 |
| miR6274-z | TTGCTATCTCCGGCCAATAAC | 21 | 5p | Chr4 | 19521466 | 19521567 | - | 102 | -36.9 | 34.31 | 1.05 |
| miR171-y | TTGAGCCGCGTCAATATCTCC | 21 | 3p | Chr4 | 22770165 | 22770285 | - | 121 | -46.6 | 39.67 | 0.97 |
| miR6266-z | TAAATGCAGGGGCAAAATGAT | 21 | 5p | Chr4 | 24363401 | 24363655 | - | 255 | -61.6 | 23.92 | 1.01 |
| miR5248-z | CTTTTCAGTTGGCATGCCTTC | 21 | 3p | Chr5 | 14823995 | 14824090 | + | 96 | -22.6 | 41.67 | 0.56 |
| miR6266-z | TAAATGCAGGGGCAAAATGAT | 21 | 5p | Chr5 | 19553759 | 19554009 | + | 251 | -51.2 | 21.91 | 0.93 |
| miR159-z | CTTGGATTGAAGGGAGCTCCA | 21 | 3p | Chr5 | 22211030 | 22211225 | + | 196 | -102.4 | 50.51 | 1.03 |
| miR166-x | GGAATGTTGTCTGGCTCGAGG | 21 | 5p | Chr5 | 22972478 | 22972649 | + | 172 | -69.8 | 39.53 | 1.03 |
| miR166-y | TCGGACCAGGCTTCATTCCCC | 21 | 3p | Chr5 | 22972478 | 22972649 | + | 172 | -69.8 | 39.53 | 1.03 |
| miR172-y | GGAATCTTGATGATGCTGCAG | 21 | 3p | Chr5 | 25787240 | 25787348 | + | 109 | -52.5 | 46.79 | 1.03 |
| miR319-x | AGAGCTTTCTTCAGTCCACTC | 21 | 5p | Chr5 | 27247976 | 27248220 | + | 245 | -86.2 | 42.04 | 0.84 |
| miR319-z | TTGGACTGAAGGGAGCTCCTC | 21 | 3p | Chr5 | 27247976 | 27248220 | + | 245 | -86.2 | 42.04 | 0.84 |
| miR166-y | TCGGACCAGGCTTCATTCCCC | 21 | 3p | Chr5 | 29880384 | 29880549 | + | 166 | -67.7 | 46.39 | 0.88 |
| miR10986-z | TGGCACCAAAGTCACCACCCG | 21 | 5p | Chr5 | 1665120 | 1665337 | - | 218 | -106.6 | 37.61 | 1.3 |
| miR6266-z | TAAATGCAGGGGCAAAATGAT | 21 | 5p | Chr5 | 3046683 | 3046937 | - | 255 | -68.7 | 25.49 | 1.06 |
| miR11544-z | TTGCAGCTGTTGTGACTCCA | 20 | 3p | Chr5 | 23497417 | 23497507 | - | 91 | -27.6 | 43.96 | 0.69 |
| miR160-x | TGCCTGGCTCCCTGTATGCCA | 21 | 5p | Chr5 | 24469138 | 24469259 | - | 122 | -52.2 | 49.18 | 0.87 |
| miR160-y | GCGTATGAGGAGCCATGCATA | 21 | 3p | Chr5 | 24469138 | 24469259 | - | 122 | -52.2 | 49.18 | 0.87 |
| miR393-x | TTCCAAAGGGATCGCATTGAT | 21 | 5p | Chr5 | 28727480 | 28727587 | - | 108 | -57.7 | 40.74 | 1.31 |
| miR828-x | TCTTGCTCAAATGAGTATTCC | 21 | 5p | Chr5 | 29752137 | 29752261 | - | 125 | -54.1 | 43.2 | 1 |
| miR828-y | TCATTTCAGCAAGCAGCGTTA | 21 | 3p | Chr5 | 29752137 | 29752261 | - | 125 | -54.1 | 43.2 | 1 |
| miR6281-z | GTTAGAGATAGAGAGAGTGAG | 21 | 5p | Chr6 | 3796572 | 3796727 | + | 156 | -84.2 | 46.79 | 1.15 |
| miR164-x | TGGAGAAGCAGGGCACGTGCA | 21 | 5p | Chr6 | 7400192 | 7400330 | + | 139 | -66.3 | 50.36 | 0.95 |
| miR164-y | CACGTGCTCCCCTTCTCCAAC | 21 | 3p | Chr6 | 7400192 | 7400330 | + | 139 | -66.3 | 50.36 | 0.95 |
| miR167-y | GGTCATGCTCTGACAGCCTCACT | 23 | 3p | Chr6 | 8914865 | 8914990 | + | 126 | -53.7 | 41.27 | 1.03 |
| miR6285-z | TAGTGAAGTTTGAATTAGGGCT | 22 | 5p | Chr6 | 12690522 | 12690602 | + | 81 | -36.8 | 39.51 | 1.15 |
| miR6300-z | GTCGTTGTAGTATAGTGGTA | 20 | 3p | Chr6 | 7715912 | 7716277 | - | 366 | -66.5 | 31.42 | 0.58 |
| miR6284-z | TTGGACCATGGATGAAGATTC | 21 | 3p | Chr6 | 7885026 | 7885124 | - | 99 | -36.4 | 49.49 | 0.74 |
| miR166-y | TCGGACCAGGCTTCATTCCCC | 21 | 3p | Chr6 | 9142053 | 9142156 | - | 104 | -53.8 | 46.15 | 1.12 |
| miR535-x | TGACAACGAGAGAGAGCACGC | 21 | 5p | Chr6 | 11379363 | 11379459 | - | 97 | -61.3 | 51.55 | 1.23 |
| miR535-z | TTGACGACGAGAGAGAGCACG | 21 | 5p | Chr6 | 11383184 | 11383282 | - | 99 | -58.5 | 50.51 | 1.17 |
| miR6288-z | AGAAAATGACAAGTGACTAGT | 21 | 3p | Chr6 | 14755058 | 14755159 | - | 102 | -50.2 | 26.47 | 1.86 |
| miR166-x | GGAATGTTGTCTGGCTCGAGG | 21 | 5p | Chr7 | 4610944 | 4611125 | + | 182 | -67.5 | 40.66 | 0.91 |
| miR166-y | TCGGACCAGGCTTCATTCCCC | 21 | 3p | Chr7 | 4610944 | 4611125 | + | 182 | -67.5 | 40.66 | 0.91 |
| miR157-x | TTGACAGAAGATAGAGAGCAC | 21 | 5p | Chr7 | 10165907 | 10166030 | + | 124 | -57.4 | 42.74 | 1.08 |
| miR157-y | GCTCTCTATGCTTCTGTCATC | 21 | 3p | Chr7 | 10165907 | 10166030 | + | 124 | -57.4 | 42.74 | 1.08 |
| miR6294-z | TGGTGTAGGCTAATCACAATC | 21 | 3p | Chr7 | 11024070 | 11024272 | + | 203 | -86.2 | 42.36 | 1 |
| miR399-x | AGGGCTTCTCTCCTTTGGCAGG | 22 | 5p | Chr7 | 847117 | 847233 | - | 117 | -59.6 | 45.3 | 1.12 |
| miR399-z | CGCCAAAGGAGAGTTGCCCTT | 21 | 3p | Chr7 | 847117 | 847233 | - | 117 | -59.6 | 45.3 | 1.12 |
| miR6275-z | AGTGGAAGTAGCAAGGGGAAG | 21 | 3p | Chr7 | 869439 | 869544 | - | 106 | -64.8 | 45.28 | 1.35 |
| miR319-x | AGAGCTTTCTTCAGTCCACTC | 21 | 5p | Chr7 | 2879839 | 2880045 | - | 207 | -85 | 41.55 | 0.99 |
| miR319-y | TTGGACTGAAGGGAGCTCCCT | 21 | 3p | Chr7 | 2879839 | 2880045 | - | 207 | -85 | 41.55 | 0.99 |
| miR6266-z | TAAATGCAGGGGCAAAATGAT | 21 | 5p | Chr7 | 4335873 | 4335990 | - | 118 | -42.7 | 24.58 | 1.47 |
| miR162-x | GGAGGCAGCGGTTCATCGATC | 21 | 5p | Chr7 | 9571603 | 9571725 | - | 123 | -44.6 | 47.15 | 0.77 |
| miR162-y | TCGATAAACCTCTGCATCCAG | 21 | 3p | Chr7 | 9571603 | 9571725 | - | 123 | -44.6 | 47.15 | 0.77 |
| miR6294-z | TGGTGTAGGCTAATCACAATC | 21 | 3p | Chr7 | 11024069 | 11024276 | - | 208 | -95 | 41.83 | 1.09 |
| miR168-x | TCGCTTGGTGCAGGTCGGGAA | 21 | 5p | Chr7 | 12794215 | 12794381 | - | 167 | -75.4 | 56.89 | 0.79 |
| miR168-y | CCCGCCTTGCATCAACTGAAT | 21 | 3p | Chr7 | 12794215 | 12794381 | - | 167 | -75.4 | 56.89 | 0.79 |
| miR11602-z | TCTAACGGAACGCTATTGGATC | 22 | 3p | Chr8 | 79346 | 79453 | + | 108 | -20.6 | 34.26 | 0.56 |
| miR827-y | TTAGATGACCATCAACAAAC | 20 | 3p | Chr8 | 1141610 | 1141717 | + | 108 | -35.1 | 27.78 | 1.17 |
| miR827-z | TTAGATGACCATCAACAAACA | 21 | 3p | Chr8 | 1141610 | 1141717 | + | 108 | -35.1 | 27.78 | 1.17 |
| miR530-x | TCTGCATTTGCACCTGCACCT | 21 | 5p | Chr8 | 8990045 | 8990156 | + | 112 | -45.7 | 41.96 | 0.97 |
| miR530-z | TGCATTTGCACCTGCACCTCT | 21 | 5p | Chr8 | 8990045 | 8990156 | + | 112 | -45.7 | 41.96 | 0.97 |
| miR396-x | TTCCACAGCTTTCTTGAACTT | 21 | 5p | Chr8 | 2290620 | 2290754 | - | 135 | -56.2 | 45.19 | 0.92 |
| miR396-y | GCTCAAGAAAGCTGTGGGAGA | 21 | 3p | Chr8 | 2290620 | 2290754 | - | 135 | -56.2 | 45.19 | 0.92 |
| miR171-z | TTGAGCCGCGCCAATATCACT | 21 | 3p | Chr8 | 2312427 | 2312535 | - | 109 | -48 | 44.04 | 1 |
| miR6279-z | TAGACAAGAATTCCAGAGACC | 21 | 3p | Chr8 | 8126805 | 8126952 | - | 148 | -30.2 | 37.84 | 0.54 |
| miR6278-z | TGAACCTTGTGTACAAATTGGC | 22 | 3p | Chr8 | 16664928 | 16665025 | - | 98 | -49 | 40.82 | 1.23 |
| miR6278-z | TGAACCTTGTGTACAAATTGGC | 22 | 3p | Chr8 | 16668672 | 16668769 | - | 98 | -45.2 | 36.73 | 1.26 |
| miR11602-z | TCTAACGGAACGCTATTGGATC | 22 | 3p | scaffold15 | 14875 | 14982 | + | 108 | -20.6 | 34.26 | 0.56 |
| miR11602-z | TCTAACGGAACGCTATTGGATC | 22 | 3p | scaffold15 | 68413 | 68520 | - | 108 | -20.6 | 34.26 | 0.56 |
| miR11602-z | TCTAACGGAACGCTATTGGATC | 22 | 3p | scaffold20 | 60663 | 60770 | + | 108 | -20.6 | 34.26 | 0.56 |
| miR11602-z | TCTAACGGAACGCTATTGGATC | 22 | 3p | scaffold20 | 114196 | 114303 | - | 108 | -20.6 | 34.26 | 0.56 |
| miR5248-z | CTTTTCAGTTGGCATGCCTTC | 21 | 5p | scaffold22 | 61199 | 61394 | + | 196 | -54.8 | 43.88 | 0.64 |
| miR5248-z | CTTTTCAGTTGGCATGCCTTC | 21 | 3p | scaffold22 | 80677 | 80991 | + | 315 | -57.3 | 33.65 | 0.54 |
| miR6274-z | TTGCTATCTCCGGCCAATAAC | 21 | 5p | scaffold30 | 40106 | 40205 | + | 100 | -41 | 41 | 1 |

Table S9. Small RNAs annotated to different categories in the degradome sequencing.

| Category | Total tags | Percentage_total | Unique tags | Percentage_unique |
| --- | --- | --- | --- | --- |
| rRNA | 632055 | 2.75% | 10964 | 0.17% |
| tRNA | 392 | 0.00% | 113 | 0.00% |
| snRNA | 530 | 0.00% | 287 | 0.00% |
| snoRNA | 16817 | 0.07% | 814 | 0.01% |
| polyN | 21936 | 0.10% | 11676 | 0.18% |
| exon_sense | 12492450 | 54.45% | 3231200 | 49.47% |
| exon_antisense | 351309 | 1.53% | 102974 | 1.58% |
| other | 9427149 | 41.09% | 3173988 | 48.59% |
| total | 22942638 | 100.00% | 6532016 | 100.00% |

Table S10. Top 20 GO biological process annotated to target genes of DEmiRs across stage comparison endodormancy I-vs-endodormancy II (A), endodormancy II-vs-ecodormancy (B), ecodormancy-vs-bud flush (C).

| Comparison | GO term | Description | P-value | FDR |
| --- | --- | --- | --- | --- |
| Endodor I-vs-Endodor II | GO:0003156 | regulation of organ formation | 3.83E-05 | 1.74E-02 |
| Endodor I-vs-Endodor II | GO:2000027 | regulation of organ morphogenesis | 7.65E-05 | 1.74E-02 |
| Endodor I-vs-Endodor II | GO:0018342 | protein prenylation | 1.90E-04 | 2.17E-02 |
| Endodor I-vs-Endodor II | GO:0097354 | prenylation | 1.90E-04 | 2.17E-02 |
| Endodor I-vs-Endodor II | GO:0048645 | organ formation | 1.31E-03 | 7.48E-02 |
| Endodor I-vs-Endodor II | GO:0009152 | purine ribonucleotide biosynthetic process | 1.52E-03 | 7.48E-02 |
| Endodor I-vs-Endodor II | GO:0006164 | purine nucleotide biosynthetic process | 1.59E-03 | 7.48E-02 |
| Endodor I-vs-Endodor II | GO:0072522 | purine-containing compound biosynthetic process | 1.89E-03 | 7.48E-02 |
| Endodor I-vs-Endodor II | GO:0044765 | single-organism transport | 1.91E-03 | 7.48E-02 |
| Endodor I-vs-Endodor II | GO:0006633 | fatty acid biosynthetic process | 1.98E-03 | 7.48E-02 |
| Endodor I-vs-Endodor II | GO:1902578 | single-organism localization | 2.05E-03 | 7.48E-02 |
| Endodor I-vs-Endodor II | GO:0000038 | very long-chain fatty acid metabolic process | 2.33E-03 | 7.48E-02 |
| Endodor I-vs-Endodor II | GO:0006631 | fatty acid metabolic process | 2.43E-03 | 7.48E-02 |
| Endodor I-vs-Endodor II | GO:0009150 | purine ribonucleotide metabolic process | 2.50E-03 | 7.48E-02 |
| Endodor I-vs-Endodor II | GO:0072330 | monocarboxylic acid biosynthetic process | 2.63E-03 | 7.48E-02 |
| Endodor I-vs-Endodor II | GO:0009260 | ribonucleotide biosynthetic process | 2.90E-03 | 7.48E-02 |
| Endodor I-vs-Endodor II | GO:0046390 | ribose phosphate biosynthetic process | 2.90E-03 | 7.48E-02 |
| Endodor I-vs-Endodor II | GO:0015988 | energy coupled proton transmembrane transport, against electrochemical gradient | 3.94E-03 | 7.48E-02 |
| Endodor I-vs-Endodor II | GO:0022603 | regulation of anatomical structure morphogenesis | 3.94E-03 | 7.48E-02 |
| Endodor I-vs-Endodor II | GO:0006605 | protein targeting | 4.13E-03 | 7.48E-02 |
| Endodor II-vs-Ecodor | GO:0009808 | lignin metabolic process | 4.21E-25 | 2.46E-22 |
| Endodor II-vs-Ecodor | GO:0009698 | phenylpropanoid metabolic process | 1.67E-14 | 4.87E-12 |
| Endodor II-vs-Ecodor | GO:0019748 | secondary metabolic process | 2.02E-09 | 3.92E-07 |
| Endodor II-vs-Ecodor | GO:0009806 | lignan metabolic process | 1.51E-05 | 1.76E-03 |
| Endodor II-vs-Ecodor | GO:0009807 | lignan biosynthetic process | 1.51E-05 | 1.76E-03 |
| Endodor II-vs-Ecodor | GO:0003156 | regulation of organ formation | 1.28E-04 | 1.24E-02 |
| Endodor II-vs-Ecodor | GO:0044710 | single-organism metabolic process | 2.11E-04 | 1.76E-02 |
| Endodor II-vs-Ecodor | GO:2000027 | regulation of organ morphogenesis | 2.55E-04 | 1.86E-02 |
| Endodor II-vs-Ecodor | GO:0009699 | phenylpropanoid biosynthetic process | 1.06E-03 | 6.89E-02 |
| Endodor II-vs-Ecodor | GO:0044699 | single-organism process | 2.35E-03 | 1.37E-01 |
| Endodor II-vs-Ecodor | GO:0048645 | organ formation | 4.25E-03 | 2.25E-01 |
| Endodor II-vs-Ecodor | GO:0044550 | secondary metabolite biosynthetic process | 5.85E-03 | 2.54E-01 |
| Endodor II-vs-Ecodor | GO:0000038 | very long-chain fatty acid metabolic process | 7.53E-03 | 2.54E-01 |
| Endodor II-vs-Ecodor | GO:0009152 | purine ribonucleotide biosynthetic process | 8.23E-03 | 2.54E-01 |
| Endodor II-vs-Ecodor | GO:0006164 | purine nucleotide biosynthetic process | 8.60E-03 | 2.54E-01 |
| Endodor II-vs-Ecodor | GO:0072522 | purine-containing compound biosynthetic process | 1.01E-02 | 2.54E-01 |
| Endodor II-vs-Ecodor | GO:0071554 | cell wall organization or biogenesis | 1.17E-02 | 2.54E-01 |
| Endodor II-vs-Ecodor | GO:0015988 | energy coupled proton transmembrane transport, against electrochemical gradient | 1.26E-02 | 2.54E-01 |
| Endodor II-vs-Ecodor | GO:0022603 | regulation of anatomical structure morphogenesis | 1.26E-02 | 2.54E-01 |
| Endodor II-vs-Ecodor | GO:0015853 | adenine transport | 1.31E-02 | 2.54E-01 |
| Ecodor-vs-Budflush | GO:0009808 | lignin metabolic process | 8.43E-18 | 7.25E-15 |
| Ecodor-vs-Budflush | GO:0009698 | phenylpropanoid metabolic process | 1.07E-08 | 4.61E-06 |
| Ecodor-vs-Budflush | GO:0019748 | secondary metabolic process | 8.53E-05 | 2.45E-02 |
| Ecodor-vs-Budflush | GO:0044710 | single-organism metabolic process | 3.61E-04 | 6.50E-02 |
| Ecodor-vs-Budflush | GO:0009806 | lignan metabolic process | 4.73E-04 | 6.50E-02 |
| Ecodor-vs-Budflush | GO:0009807 | lignan biosynthetic process | 4.73E-04 | 6.50E-02 |
| Ecodor-vs-Budflush | GO:0008643 | carbohydrate transport | 5.29E-04 | 6.50E-02 |
| Ecodor-vs-Budflush | GO:0003156 | regulation of organ formation | 1.30E-03 | 1.39E-01 |
| Ecodor-vs-Budflush | GO:0003002 | regionalization | 2.12E-03 | 2.00E-01 |
| Ecodor-vs-Budflush | GO:0050826 | response to freezing | 2.56E-03 | 2.00E-01 |
| Ecodor-vs-Budflush | GO:2000027 | regulation of organ morphogenesis | 2.56E-03 | 2.00E-01 |
| Ecodor-vs-Budflush | GO:0048645 | organ formation | 3.44E-03 | 2.47E-01 |
| Ecodor-vs-Budflush | GO:0048507 | meristem development | 5.28E-03 | 3.34E-01 |
| Ecodor-vs-Budflush | GO:0009955 | adaxial/abaxial pattern specification | 5.87E-03 | 3.34E-01 |
| Ecodor-vs-Budflush | GO:0018342 | protein prenylation | 6.22E-03 | 3.34E-01 |
| Ecodor-vs-Budflush | GO:0097354 | prenylation | 6.22E-03 | 3.34E-01 |
| Ecodor-vs-Budflush | GO:0009933 | meristem structural organization | 7.92E-03 | 3.55E-01 |
| Ecodor-vs-Budflush | GO:0042126 | nitrate metabolic process | 7.99E-03 | 3.55E-01 |
| Ecodor-vs-Budflush | GO:2001057 | reactive nitrogen species metabolic process | 7.99E-03 | 3.55E-01 |
| Ecodor-vs-Budflush | GO:0048532 | anatomical structure arrangement | 8.26E-03 | 3.55E-01 |

Table S11. Top 20 KEGG pathways annotated to target genes of DEmiRs across stage comparison endodormancy I-vs-endodormancy II (A), endodormancy II-vs-ecodormancy (B), ecodormancy-vs-bud flush (C).

| Comparison | KO_ID | Descrption | Pvalue | FDR |
| --- | --- | --- | --- | --- |
| Endodor I-vs-Endodor II | ko03430 | Mismatch repair | 9.07E-03 | 1.22E-01 |
| Endodor I-vs-Endodor II | ko03030 | DNA replication | 1.42E-02 | 1.22E-01 |
| Endodor I-vs-Endodor II | ko00900 | Terpenoid backbone biosynthesis | 1.48E-02 | 1.22E-01 |
| Endodor I-vs-Endodor II | ko00592 | alpha-Linolenic acid metabolism | 1.78E-02 | 1.22E-01 |
| Endodor I-vs-Endodor II | ko03420 | Nucleotide excision repair | 2.04E-02 | 1.22E-01 |
| Endodor I-vs-Endodor II | ko00190 | Oxidative phosphorylation | 2.55E-02 | 1.28E-01 |
| Endodor I-vs-Endodor II | ko03440 | Homologous recombination | 4.32E-02 | 1.85E-01 |
| Endodor I-vs-Endodor II | ko00511 | Other glycan degradation | 6.34E-02 | 2.23E-01 |
| Endodor I-vs-Endodor II | ko00591 | Linoleic acid metabolism | 6.70E-02 | 2.23E-01 |
| Endodor I-vs-Endodor II | ko00010 | Glycolysis / Gluconeogenesis | 7.42E-02 | 2.23E-01 |
| Endodor I-vs-Endodor II | ko03410 | Base excision repair | 1.43E-01 | 3.56E-01 |
| Endodor I-vs-Endodor II | ko04130 | SNARE interactions in vesicular transport | 1.43E-01 | 3.56E-01 |
| Endodor I-vs-Endodor II | ko00071 | Fatty acid degradation | 1.66E-01 | 3.56E-01 |
| Endodor I-vs-Endodor II | ko03020 | RNA polymerase | 1.66E-01 | 3.56E-01 |
| Endodor I-vs-Endodor II | ko00350 | Tyrosine metabolism | 2.13E-01 | 3.78E-01 |
| Endodor I-vs-Endodor II | ko00562 | Inositol phosphate metabolism | 2.13E-01 | 3.78E-01 |
| Endodor I-vs-Endodor II | ko01110 | Biosynthesis of secondary metabolites | 2.14E-01 | 3.78E-01 |
| Endodor I-vs-Endodor II | ko04070 | Phosphatidylinositol signaling system | 2.41E-01 | 4.01E-01 |
| Endodor I-vs-Endodor II | ko00460 | Cyanoamino acid metabolism | 2.75E-01 | 4.25E-01 |
| Endodor I-vs-Endodor II | ko00230 | Purine metabolism | 2.87E-01 | 4.25E-01 |
| Endodor II-vs-Ecodor | ko00950 | Isoquinoline alkaloid biosynthesis | 6.57E-09 | 2.89E-07 |
| Endodor II-vs-Ecodor | ko00350 | Tyrosine metabolism | 2.74E-07 | 6.03E-06 |
| Endodor II-vs-Ecodor | ko00515 | Mannose type O-glycan biosyntheis | 7.49E-03 | 1.10E-01 |
| Endodor II-vs-Ecodor | ko00030 | Pentose phosphate pathway | 5.35E-02 | 5.66E-01 |
| Endodor II-vs-Ecodor | ko01100 | Metabolic pathways | 6.90E-02 | 5.66E-01 |
| Endodor II-vs-Ecodor | ko00511 | Other glycan degradation | 1.20E-01 | 5.66E-01 |
| Endodor II-vs-Ecodor | ko00591 | Linoleic acid metabolism | 1.27E-01 | 5.66E-01 |
| Endodor II-vs-Ecodor | ko00190 | Oxidative phosphorylation | 1.32E-01 | 5.66E-01 |
| Endodor II-vs-Ecodor | ko04075 | Plant hormone signal transduction | 1.36E-01 | 5.66E-01 |
| Endodor II-vs-Ecodor | ko00073 | Cutin, suberine and wax biosynthesis | 1.65E-01 | 5.66E-01 |
| Endodor II-vs-Ecodor | ko00780 | Biotin metabolism | 1.72E-01 | 5.66E-01 |
| Endodor II-vs-Ecodor | ko00920 | Sulfur metabolism | 2.14E-01 | 5.66E-01 |
| Endodor II-vs-Ecodor | ko00010 | Glycolysis / Gluconeogenesis | 2.21E-01 | 5.66E-01 |
| Endodor II-vs-Ecodor | ko00513 | Various types of N-glycan biosynthesis | 2.26E-01 | 5.66E-01 |
| Endodor II-vs-Ecodor | ko04712 | Circadian rhythm - plant | 2.32E-01 | 5.66E-01 |
| Endodor II-vs-Ecodor | ko00909 | Sesquiterpenoid and triterpenoid biosynthesis | 2.38E-01 | 5.66E-01 |
| Endodor II-vs-Ecodor | ko03430 | Mismatch repair | 2.49E-01 | 5.66E-01 |
| Endodor II-vs-Ecodor | ko01110 | Biosynthesis of secondary metabolites | 2.53E-01 | 5.66E-01 |
| Endodor II-vs-Ecodor | ko00906 | Carotenoid biosynthesis | 2.61E-01 | 5.66E-01 |
| Endodor II-vs-Ecodor | ko00520 | Amino sugar and nucleotide sugar metabolism | 2.66E-01 | 5.66E-01 |
| Ecodor-vs-Budflush | ko00950 | Isoquinoline alkaloid biosynthesis | 1.54E-06 | 1.08E-04 |
| Ecodor-vs-Budflush | ko00350 | Tyrosine metabolism | 5.31E-05 | 1.86E-03 |
| Ecodor-vs-Budflush | ko00603 | Glycosphingolipid biosynthesis - globo and isoglobo series | 8.03E-04 | 1.65E-02 |
| Ecodor-vs-Budflush | ko01100 | Metabolic pathways | 1.03E-03 | 1.65E-02 |
| Ecodor-vs-Budflush | ko00604 | Glycosphingolipid biosynthesis - ganglio series | 1.51E-03 | 1.65E-02 |
| Ecodor-vs-Budflush | ko00531 | Glycosaminoglycan degradation | 1.60E-03 | 1.65E-02 |
| Ecodor-vs-Budflush | ko00592 | alpha-Linolenic acid metabolism | 1.65E-03 | 1.65E-02 |
| Ecodor-vs-Budflush | ko01110 | Biosynthesis of secondary metabolites | 2.54E-03 | 2.23E-02 |
| Ecodor-vs-Budflush | ko01040 | Biosynthesis of unsaturated fatty acids | 4.37E-03 | 3.40E-02 |
| Ecodor-vs-Budflush | ko00970 | Aminoacyl-tRNA biosynthesis | 6.17E-03 | 4.23E-02 |
| Ecodor-vs-Budflush | ko00071 | Fatty acid degradation | 6.64E-03 | 4.23E-02 |
| Ecodor-vs-Budflush | ko00860 | Porphyrin and chlorophyll metabolism | 1.30E-02 | 7.60E-02 |
| Ecodor-vs-Budflush | ko00410 | beta-Alanine metabolism | 1.55E-02 | 8.07E-02 |
| Ecodor-vs-Budflush | ko00515 | Mannose type O-glycan biosyntheis | 1.61E-02 | 8.07E-02 |
| Ecodor-vs-Budflush | ko00940 | Phenylpropanoid biosynthesis | 2.91E-02 | 1.31E-01 |
| Ecodor-vs-Budflush | ko00511 | Other glycan degradation | 2.99E-02 | 1.31E-01 |
| Ecodor-vs-Budflush | ko00900 | Terpenoid backbone biosynthesis | 4.39E-02 | 1.79E-01 |
| Ecodor-vs-Budflush | ko00640 | Propanoate metabolism | 4.62E-02 | 1.79E-01 |
| Ecodor-vs-Budflush | ko00563 | Glycosylphosphatidylinositol(GPI)-anchor biosynthesis | 5.23E-02 | 1.87E-01 |
| Ecodor-vs-Budflush | ko00051 | Fructose and mannose metabolism | 5.33E-02 | 1.87E-01 |
